# Genome-wide survey and expression analysis of Dof transcription factor family in sweetpotato shed light on their promising functions in stress tolerance

**DOI:** 10.3389/fpls.2023.1140727

**Published:** 2023-02-21

**Authors:** Chengbin Zhang, Tingting Dong, Jing Yu, Haiting Hong, Siyuan Liu, Fen Guo, Hongting Ma, Jianling Zhang, Mingku Zhu, Xiaoqing Meng

**Affiliations:** ^1^ Institute of Integrative Plant Biology, School of Life Sciences, Jiangsu Normal University, Xuzhou, China; ^2^ Laboratory of Plant Germplasm Innovation and Utilization, School of Life Sciences, Liaocheng University, Liaocheng, China

**Keywords:** abiotic stress, Dof transcription factor, expression analysis, molecular characterization, sweetpotato

## Abstract

DNA-binding with one finger (Dof) transcription factors play a crucial role in plant abiotic stress regulatory networks, although massive Dofs have been systematically characterized in plants, they have not been identified in the hexaploid crop sweetpotato. Herein, 43 *IbDof* genes were detected to be disproportionally dispersed across 14 of the 15 chromosomes of sweetpotato, and segmental duplications were discovered to be the major driving force for the expansion of *IbDofs*. The collinearity analysis of *IbDofs* with their related orthologs from eight plants revealed the potential evolutionary history of Dof gene family. Phylogenetic analysis displayed that IbDof proteins were assigned into nine subfamilies, and the regularity of gene structures and conserved motifs was consistent with the subgroup classification. Additionally, five chosen *IbDof* genes were shown to be substantially and variably induced under various abiotic conditions (salt, drought, heat, and cold), as well as hormone treatments (ABA and SA), according to their transcriptome data and qRT-PCR experiments. Consistently, the promoters of *IbDofs* contained a number of cis-acting elements associated with hormone and stress responses. Besides, it was noted that IbDof2 had transactivation activity in yeasts, while IbDof-11/-16/-36 did not, and protein interaction network analysis and yeast two-hybrid experiments revealed a complicated interaction connection amongst IbDofs. Collectively, these data lay a foundation for further functional explorations of *IbDof* genes, especially with regards to the possible application of multiple IbDof members in breeding the tolerant plants.

## Introduction

Sweetpotato is an important root tuber crop with effective output of more than 100 million tons per annum. In terms of starch staple food, sweetpotato is second only to cassava in number in East Africa (Ssamula et al., 2020). Asia accounts not only for more than 50% of sweetpotato-producing region in the world, but also for approximately 80% of the world production (Liu, 2017). In developed nations, sweetpotato was initially grown for fresh consumption or preserved food, while the market for sweetpotato as a bio-based industrial food or value-added products is growing by degrees. Additionally, sweetpotato can also be used as suitable raw materials for industrial productions, such as for the production of bioenergy, including ethanol and butanol (Ziska et al., 2009; Jin et al., 2022). Nevertheless, adverse abiotic stresses such as salt, drought and cold severely limit crop growth, development and yield (Corrales et al., 2017; Meng et al., 2023). Genetic engineering is widely proven to be a powerful way to improve crop resistance, among them, transcription factors (TFs) are promising and efficient members to solve the problems encountered by plants in adversity, such as AP2/ERF, NAC, WRKY, MYB, GRAS and Dof TF families (Gupta et al., 2015; Erpen et al., 2018; Ichimaru et al., 2022; Zhang et al., 2022).

Typically, plant-specific Dof TFs contain a conserved Dof domain located at the N-terminal region with a highly conserved zinc finger motif, which can specifically bind to DNA sequences with a 5’-AAAG-3’ core in the promoters of the target genes. Additionally, the C-terminal of Dof TFs include a highly variable transcriptional regulation region and a nuclear localization signal (Gupta et al., 2015; Waqas et al., 2020). Dof domain consists of 50–52 amino acids with four conserved cysteines (CX_2_CX_21_CX_2_C), which can form a single zinc finger, hence it is called DNA-binding with one finger (Waqas et al., 2020). The Dof domain is considered as a bifunctional region: DNA-protein and protein-protein interactions (Krohn et al., 2002; Gupta et al., 2015). The first Dof gene, *ZmDof1*, was found in maize (Yanagisawa and Izui, 1993), and gradually expended into diverse taxonomic groups ranging from ferns and mosses to vascular plants (Kushwaha et al., 2011; Lohani et al., 2021). Presently, genome-wide isolation of Dof TFs are emerging from sundry plants, for example, 30, 30, 119, 74 and 114 Dofs were found in monocots such as *Oryza sativa* (Khan et al., 2021), *Sorghum bicolor* (Xiao et al., 2022), *Saccharum officinarum* (Gupta et al., 2014), *Musa nana* (Dong et al., 2016), and *Gossypium hirsutum* (Li et al., 2018), respectively. And there are 36, 117, 33, 34, 33 and 29 Dof members were found in eudicots like *Arabidopsis thaliana* (Lijavetzky et al., 2003), *Brassica napus* (Lohani et al., 2021), *Piper Nigrum* (Kang et al., 2016), *Solanum lycopersicum* (Cai et al., 2013), *Capsicum annuum* (Wu et al., 2016), and *Solanum melongena* (Wei et al., 2018), respectively.

Dof proteins have been substantially evidenced to partake gene expression in diverse processes such as seed maturation and germination, seedling development, light response, carbon and nitrogen metabolism, leaf axial patterning, as well as metabolic pathways involving hormones such as salicylic acid and response (Gupta et al., 2015; Ruta et al., 2020). For instance, Dof TFs VASCULAR-RELATED DOF1 (VDOF1) and VDOF2 were reported to regulate vascular cell differentiation and lignin biosynthesis in Arabidopsis (Ramachandran et al., 2020). The Arabidopsis Dof TF CDF3 was involved in nitrogen responses and improved nitrogen use efficiency in transgenic tomato (Corrales et al., 2017). Additionally, multiple studies have revealed that Dof TFs participate in the response to various environmental changes (Corrales et al., 2017; Waqas et al., 2020). For example, the transcription of tomato *SlCDF1–5* (Cycling Dof Factors) genes was significantly induced under osmotic, salt, heat, and low-temperature stresses, overexpression of *SlCDF1* or *SlCDF3* in Arabidopsis both showed improved drought and salt tolerance (Corrales et al., 2014). Rice *OsDOF15* was found to be a salt-responsive gene which participated in salt-induced inhibition of primary root growth (Qin et al., 2019). The expression of Arabidopsis *CDF3* is highly induced by drought, extreme temperature and ABA treatments. The *cdf3* mutant is more sensitive to drought and cold stress, whereas enhanced tolerance to drought, cold and osmotic stress was detected in *CDF3*-overexpressing plants (Corrales et al., 2017). Besides, *Tamarix hispida* ThDof TF functions as an upstream regulator of TheIF1A that plays a positive factor in plant salt and osmotic stress tolerance *via* the regulation of associated enzymes and ROS scavenging (Yang et al., 2017a). And *Juglans regia* JrDof3 TF also contributed to improve the high temperature stress response of JrGRAS2, which could effectively regulate the expression of *HSPs* to enhance high temperature stress tolerance (Yang et al., 2018). Similarly, grape *VaDof17d* gene was recently reported to be positively correlated with cold tolerance (Wang et al., 2021). Therefore, Dof TFs are one of the pivotal factors to enhance abiotic stress tolerance in plants by genetic engineering. Nevertheless, despite this, the specific roles of most Dof TFs in plants remain unknown, and further studies are still imperative to explore the regulatory pathways for abiotic stress tolerance through Dof TFs.

The recent advancements in plant genomic sequencing have significantly accelerated the identification of Dof genes in various plants that will expand our understandings of their potential functions in plant stress response. However, the genome-wide structure and function research of most Dofs remain to be elucidated, especially in the important hexaploid crop sweetpotato. Although the genome of hexaploid sweetpotato has been sequenced more than 5 years (Yang et al., 2017b), the identification and functional analysis of the sweetpotato Dof TF family is still blank. Here, genome-wide and transcriptome-based analysis of *IbDof* genes in sweetpotato were accomplished, including chromosomal location, motif composition, gene structure, conserved domain, collinearity analysis and protein-protein interaction. And the expression analysis of *IbDof* genes under various abiotic stresses and hormones shed light on their promising functions in stress tolerance. The information gained here will be significant for laying the groundwork for the functional characterization of multiple valuable sweetpotato IbDof TFs in response to abiotic stress.

## Materials and methods

### Identification of putative *IbDof* genes in sweetpotato genomes

The completed genome sequence and GFF annotation data of sweetpotato (*Ipomoea batatas*, Taizhong6) were obtained from Ipomoea Genome Hub database (https://ipomoea-genome.org) as described in our previous report (Liu et al., 2022). The nucleotide and protein sequences of 36 Dof TFs of *Arabidopsis thaliana* and 30 Dof TFs of *Oryza sativa* (**Supplementary Table S1**) were downloaded from TAIR (https://www.arabidopsis.org/) and Rice Genome Annotation Project (http://rice.uga.edu/), respectively. Then the acquired Dof protein sequences of rice and Arabidopsis were used as query sequences for BLASTP program (E-value ≤ 1e-5) with 500 MumofHits and 250 MumoAligns to search all the possible Dof members in sweetpotato by Tbtools (Chen et al., 2020). Ultimately, 44 non-redundant protein sequences were obtained, and the Pfam database (http://pfam.xfam.org/), online batch CD-search program (https://www.ncbi.nlm.nih.gov/cdd/Structure/cdd/wrpsb.cgi) and PROSITE database (https://prosite.expasy.org/) were used to exclude the proteins without the Dof domain. All the sequence information of 43 candidate *IbDof* genes was listed in **Supplementary Table S2**.

### Analysis of physicochemical characteristics of IbDof proteins

The ExPASy tool (http://expasy.org/) was used to predict the specific isoelectric point and molecular weight of each IbDof protein. The phosphorylation sites of IbDofs were detected using NetPhos 3.1 (http://www.cbs.dtu.dk/services/NetPhos/). To estimate their subcellular locations, the Plant-mPLoc software (http://www.csbio.sjtu.edu.cn/bioinf/plant-multi/) was used. Tbtools software was used to create the intron-exon architecture of the *IbDof* genes by matching their coding sequences and genomic sequences (Chen et al., 2020). In order to identify any potential cis-elements related to hormones and stresses, the 2.0 kb promoter sections of 43 *IbDof* genes were taken from the Ipomoea Genome Hub and were uploaded to the plantCARE database (http://bioinformatics.psb.ugent.be/webtools/plantcare/html/) for detection.

### Analysis of phylogenetic relationship, conserved domain, and protein interaction network

The amino acid sequences of 43 IbDofs in sweetpotato and the well-classified AtDof proteins in Arabidopsis and OsDof proteins in rice (Lijavetzky et al., 2003) were utilized to build the unrooted phylogenetic tree using the Maximum Likelihood method by MEGA-X software (Kumar et al., 2018). All the Dof protein sequences were first aligned by ClustalW with default settings, then the following parameters were used to generate the phylogenetic relationship of 43 IbDofs: the best evolutionary model of JTT + G + I + F, a bootstrap value of 1000 with partial deletions. Based on the previously defined parameters in Arabidopsis and rice, MEME 5.3.3 software (https://meme-suite.org/meme/tools/meme) was used to construct the conserved motifs with a maximum number of 15, and a length of > 6 and < 200 amino acids (Khan et al., 2021). The possible protein interaction network was carried out using STRING 11.0 (https://string-db.org/).

### Chromosome position and collinearity analysis of *IbDof* genes in sweetpotato

According to the GFF annotations of the sweetpotato genomes, 43 *IbDof* genes were linked with the chromosomes. In order to conduct a synteny analysis between *IbDofs* and genes from other plant species, the genome sequence and annotation data of sweetpotato and eight representative species (including *Ipomoea triloba*, *Ipomoea trifida*, Arabidopsis, rice, tomato, pepper, cabbage, and *Brassica oleracea*), were downloaded from various databases such as Ipomoea Genome Hub, TAIR, Ensembl (http://plants.ensembl.org/index.html), and Phytozome (https://phytozome.jgi.doe.gov/pz/portal.html). MCScanX software was used to evaluate the association of gene duplication and collinearity using default settings. Circos and TBtools softwares were used to display the obtained results, and the minimum block size setting is 30 (Krzywinski et al., 2009; Chen et al., 2020; Guo et al., 2022).

### Screening of salt-responsive *IbDof* genes based on RNA-seq analysis

After treatment with 150 mM NaCl for 24 hours, the adventitious roots of two substantially different sweetpotato cultivars XuShu 22 (salt-tolerant) and XuShu 32 (salt-sensitive) were collected for transcriptome analysis. Read counts were used to measure gene expression and differentially expressed genes were screened by false discovery rate (FDR) and Log2 (fold change) as previously described (Meng et al., 2020b). Then gene functions were annotated based on the information from several databases such as the sweetpotato reference genome database, Nr, Pfam, and SwissProt. The salt-stress responsive or genotype-specific *IbDof* genes are listed in **Supplementary Table S3**.

### Abiotic stress and hormone treatments of sweetpotato seedlings and qRT-PCR analysis

The salt-tolerant XuShu 22 seedlings were grown in 1/4 Hoagland solutions and used for all the stress and hormone treatments with three different biological replicates. The roots were immersed in 150 mM NaCl and 20% PEG6000 solutions, respectively, for salt and dehydration stress treatment, then root samples were collected. Seedlings were incubated at 4°C and 42°C, respectively, for cold and heat conditions, then leaves samples were harvested. And the leaves of seedlings were collected after spraying with 0.1 mM ABA or 2 mM SA solutions for phytohormone tests (Meng et al., 2018). All of the samples were collected at 0, 1, 12, 24, and 48 hours after each treatment.

Total RNA was extracted using RNA Extraction Kits (TianGen, Beijing, China) following the manufacturer’s instructions. PrimeScript reverse transcriptase with the gDNA Eraser (TaKaRa, Dalian, China) was used to reverse-transcribe 1 μg of each RNA sample. qRT-PCR tests were conducted using the CFX96TM Real-Time System as previously described (Bio-Rad, USA) (Meng et al., 2020a), and the sweetpotato *ARF* gene (JX177359) was employed as an internal control (Park et al., 2012). All the qRT-PCR primers are provided in **Supplementary Table S4**.

### Analysis of transactivation activity and protein interaction of IbDof proteins in yeasts

The open reading frame sequences of *IbDof-2/-11/-16/-36* genes were independently cloned into the pGBKT7 and pGADT7 vector, respectively, using the homologous recombination method. Then Y2HGold yeasts were transformed with the pGBKT7 control, recombined pGBKT7-IbDof plasmids, or both recombined pGBKT7-IbDof and pGADT7-IbDof vectors, respectively, as previously described (Zhang et al., 2022). The yeast dilution was dropped on SD/-Trp (SDO), SD/-Trp-His-Ade (TDO) medium with or without 200 ng/mL AbA (Aureobasidin A) for the transactivation detection, and the dilutions were dropped on SD/-Trp-Leu (DDO), SD/-Trp-Leu-His-Ade (QDO) medium with or without 200 ng/mL AbA for the purpose of detecting protein interactions. All of the plates were grown at 30°C to test for transactivation or protein interaction for three days. The primers used for gene cloning and vector construction are shown in **Supplementary Table S5**.

### Statistical analysis

The experiments were performed in three biological replicates. A cut-off value of two-fold for differential gene expression was established in consideration of the biological importance (Zhang et al., 2022). Graphs were produced using the SAS Institute’s OriginPro 8 program.

## Results

### Identification and characterization of the Dof gene family in sweetpotato genome

In this study, the BLASTP program was used to screen all the potential sweetpotato Dofs using the known Dof proteins from Arabidopsis and rice as query sequences. One of the proteins was, however, disqualified because it lacked the Dof domain. The remaining 43 genes were named as *IbDof1* to *IbDof43* according to the location order of sweetpotato chromosomes from top to bottom. Subsequently, the amino acid (aa) length, molecular weight (Mw), and theoretical isoelectric point (pI) of all IbDof proteins were analyzed (**Table 1**). The amino acid lengths of IbDofs range from 110 (IbDof13) to 959 (IbDof20). As a result, their MW ranges from 12.4 kDa (IbDof13) to 106.1 kDa (IbDof20), while their pI ranges from 4.94 (IbDof29) to 9.73 (IbDof43). The prediction of subcellular localization suggests that all IbDof TFs are localized in the nucleus. Besides, the IbDofs have multiple possible phosphorylation sites, ranging from 11 (IbDof27) to 102 (IbDof20), and roughly 89% of the IbDofs contain at least 20 phosphorylation sites.

**Table 1:** Characteristics of *IbDof* genes in *Ipomoea batatas*.

Gene name	Gene ID	Length	MW (Da)	PI	Subcellular location	No. of phosphorylation sites			
						Ser sites	Tyr sites	Thr sites	Total
*IbDof1*	g563.t1	269	29567.12	5.03	Nucleus	16	4	9	29
*IbDof2*	g909.t1	275	29902.17	7.17	Nucleus	26	4	6	36
*IbDof3*	g1131.t1	262	27645.86	9.18	Nucleus	26	3	7	36
*IbDof4*	g1308.t1	529	58136.22	6.19	Nucleus	41	2	13	56
*IbDof5*	g2786.t1	287	31077.49	6.19	Nucleus	18	2	13	33
*IbDof6*	g4774.t1	282	30986.35	6.78	Nucleus	21	7	7	35
*IbDof7*	g4854.t1	319	33797.46	9.2	Nucleus	27	2	13	42
*IbDof8*	g4893.t1	320	33861.58	9.2	Nucleus	27	3	14	44
*IbDof9*	g10108.t1	268	29426.78	8.22	Nucleus	17	5	10	32
*IbDof10*	g12187.t1	469	51472.98	9.58	Nucleus	32	4	6	42
*IbDof11*	g12244.t1	439	47269.37	7.11	Nucleus	48	5	15	68
*IbDof12*	g17872.t1	227	24647.6	9.25	Nucleus	16	1	6	23
*IbDof13*	g19058.t1	110	12439.99	9.66	Nucleus	7	3	2	12
*IbDof14*	g19324.t1	337	36307.7	8.21	Nucleus	43	5	7	55
*IbDof15*	g19438.t1	336	36213.6	8.21	Nucleus	41	5	5	51
*IbDof16*	g20269.t1	313	33258.39	9.48	Nucleus	18	4	4	26
*IbDof17*	g24282.t1	338	35097.92	9.39	Nucleus	34	2	12	48
*IbDof18*	g27012.t1	253	27431.46	8.15	Nucleus	26	3	7	36
*IbDof19*	g29805.t1	323	34658.06	8.44	Nucleus	36	2	5	43
*IbDof20*	g29939.t1	959	106122.5	6.77	Nucleus	80	9	13	102
*IbDof21*	g30074.t1	514	55813.84	5.28	Nucleus	40	3	19	62
*IbDof22*	g32116.t1	466	50705.95	5.76	Nucleus	53	4	19	76
*IbDof23*	g33760.t1	350	38282.31	6.93	Nucleus	36	5	11	52
*IbDof24*	g34515.t1	279	30234.75	6.15	Nucleus	22	3	12	37
*IbDof25*	g34535.t1	350	37725.58	8.9	Nucleus	33	3	9	45
*IbDof26*	g34611.t1	174	19098.54	8.61	Nucleus	8	2	2	12
*IbDof27*	g34661.t1	174	19155.6	8.61	Nucleus	7	2	2	11
*IbDof28*	g39250.t1	219	23949	8.84	Nucleus	17	3	8	28
*IbDof29*	g42238.t1	269	29776.11	4.94	Nucleus	24	2	6	32
*IbDof30*	g42376.t1	269	29762.09	4.94	Nucleus	24	3	6	33
*IbDof31*	g42798.t1	802	90431.97	7.93	Nucleus	64	10	26	100
*IbDof32*	g45550.t1	287	30912.28	6.18	Nucleus	18	2	13	33
*IbDof33*	g45578.t1	297	31961.5	6.35	Nucleus	18	2	12	32
*IbDof34*	g48073.t1	262	28714.99	8.64	Nucleus	23	7	3	33
*IbDof35*	g53151.t1	239	25725.87	9.32	Nucleus	16	3	5	24
*IbDof36*	g53369.t1	388	42412.24	9.33	Nucleus	30	4	17	51
*IbDof37*	g55838.t1	324	33387.8	9.6	Nucleus	35	3	9	47
*IbDof38*	g59149.t1	313	34076.93	9.33	Nucleus	40	2	9	51
*IbDof39*	g60025.t1	388	42282.36	9.05	Nucleus	32	3	8	43
*IbDof40*	g60438.t1	210	22064.56	8.16	Nucleus	12	2	3	17
*IbDof41*	g63457.t1	166	19305.01	9.73	Nucleus	7	5	5	17
*IbDof42*	g63711.t1	202	22537.16	8.45	Nucleus	19	7	6	32
*IbDof43*	g63743.t1	192	21594.34	9.73	Nucleus	16	3	8	27

Length, Amino acid length; MW, Molecular weight; pl, Isoelectric point

### Chromosome distribution of sweetpotato *IbDof* genes

**Figure 1:**
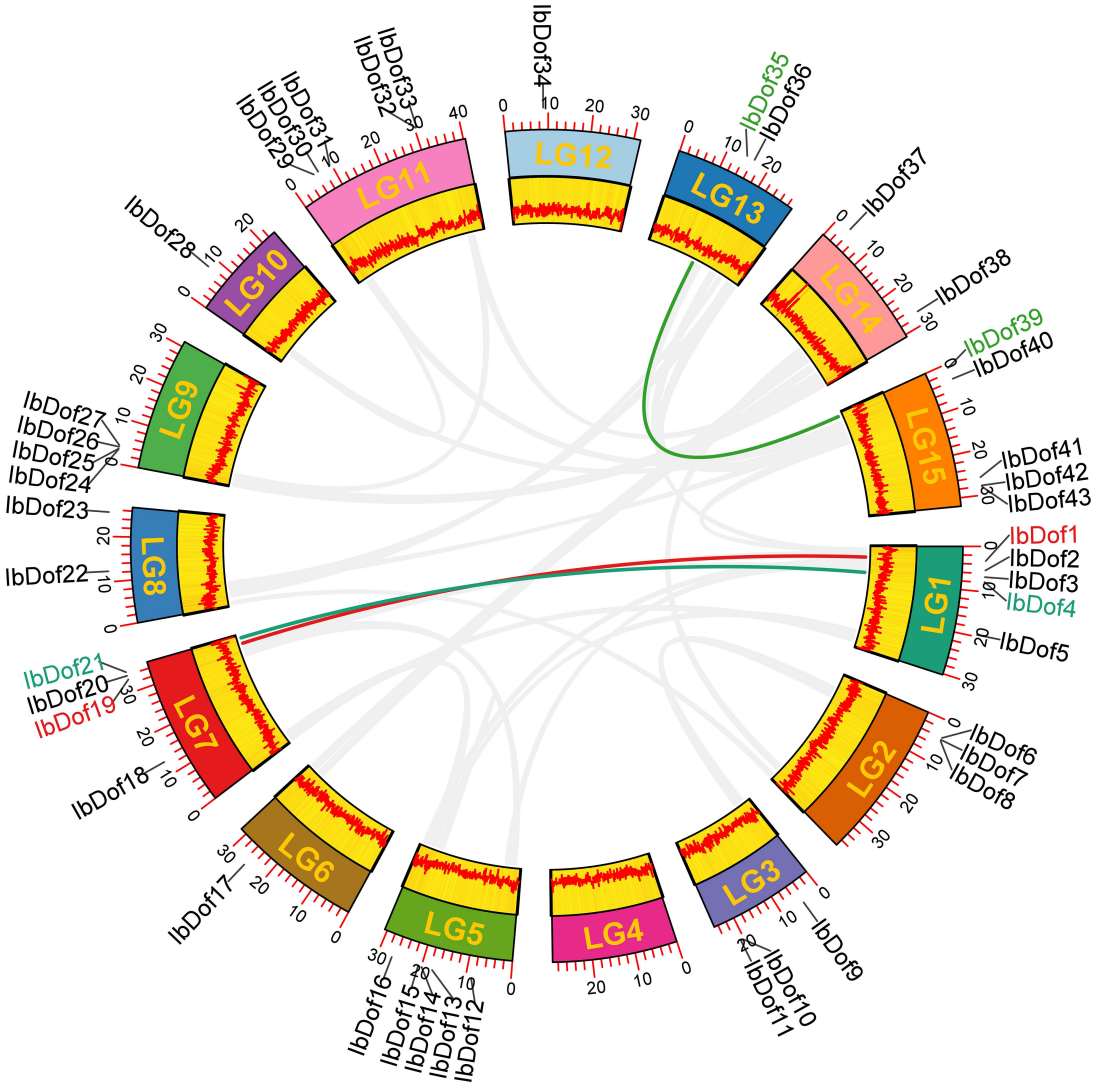
Chromosome distribution and synteny analysis of *IbDof* genes in sweetpotato chromosomes. Chromosomes LG1-LG15 are marked with colored rectangles, the location of each *IbDof* is represented by a short black line, the colored lines represent segmentally duplicated *IbDof* genes.

Physical locations determined using the GFF3 genome annotations of sweetpotato revealed that 43 *IbDof* genes were mapped to all the 15 chromosomes (Chr) except Chr 4 (**Figure 1** and **Supplementary Figure S1**). Among them, the most abundant *IbDof* genes are found on Chr 1 and Chr 5, both have five members. However, only one *IbDof* gene is present on Chr 6, Chr 10 and Chr12, and no *IbDof* gene was found on Chr 4. In the remaining chromosomes, the number of *IbDof* genes ranges from two to four. The results demonstrated that the distribution of *IbDof* gene varied greatly and was not related to chromosomal length. For instance, the short chromosome (Chr 3) has three *IbDof* genes while the long chromosome (Chr 12) only has one.

Besides, to detect the possible relationship among the 43 *IbDof* genes, the collinear analysis was conducted by the BlastP and MCScanX programs, however, no tandem repeat events were observed among the *IbDof* genes. And three gene pairs with segmental duplications were found as follows: *IbDof4*-*IbDof21*, *IbDof1*-*IbDof19*, and *IbDof35-IbDof39* (**Figure 1** and **Supplementary Table S6**). The data suggest that segmental duplications of *IbDof* genes have a primary contribution to their expansions.

### Phylogenetic relationships of IbDof proteins in sweetpotato

**Figure 2:**
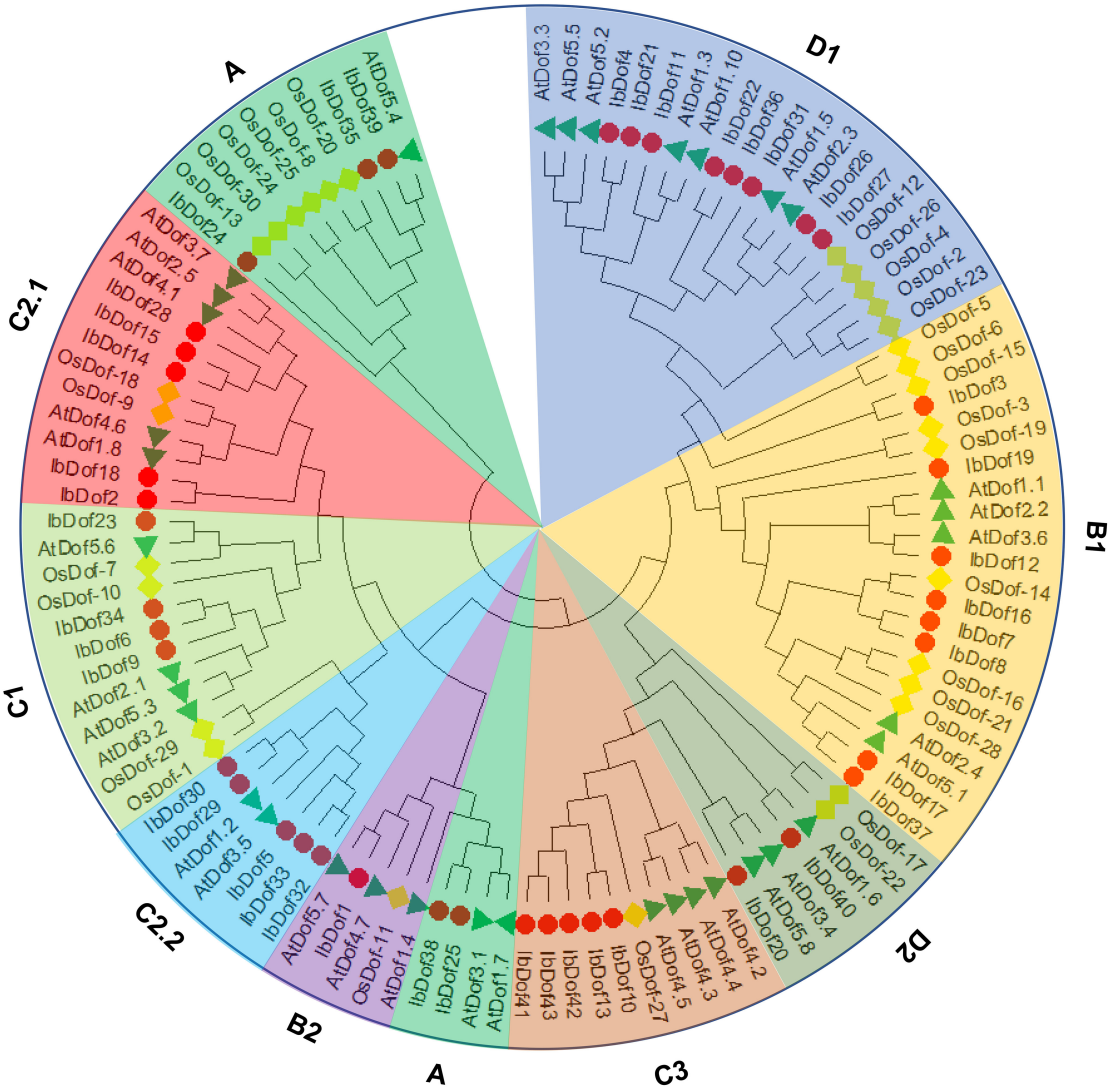
Unrooted phylogenetic tree constructed with sweetpotato IbDofs, Arabidopsis AtDofs and rice OsDofs. All the protein sequences were aligned by ClustalW and the phylogenetic relationships were derived through the Maximum Likelihood method, the best evolutionary model JTT + G + I + F calculated through MEGA X was selected with the bootstrap value of 1000. Different subgroups are named based on the reports in Arabidopsis and rice and are distinguished with different colors. The protein names are marked at the end of the branch, the red circle, green triangle and blue rhombus represent the sweetpotato IbDofs, Arabidopsis AtDofs, and rice OsDofs, respectively.

To examine the evolutionary connections of IbDofs in sweetpotato, a phylogenetic tree was created using the full amino acid sequences of 43 IbDofs, 36 Arabidopsis AtDofs and 30 rice OsDofs by Maximum Likelihood method using MEGA-X software. Previously, 36 AtDofs and 30 OsDofs were divided into nine subgroups (A, B1, B2, C1, C2.1, C2.2, C3, D1 and D2) (Lohani et al., 2021; Nan et al., 2021). Accordingly, 43 IbDofs were also divided into nine subgroups, and the sizes of each subgroup varied substantially as shown by the phylogenetic analysis (**Figure 2**). B1 and D1 subgroups had the most IbDof proteins (8 members, 18.6%), followed by A, C2.1, C2.2, and C3 subgroups (5 members, 11.6%), C1 subgroup (4 members, 9.3%), and B1 subgroup (1 member, 2.3%).

### Gene structure, conserved domain and motif analysis of IbDofs

**Figure 3:**
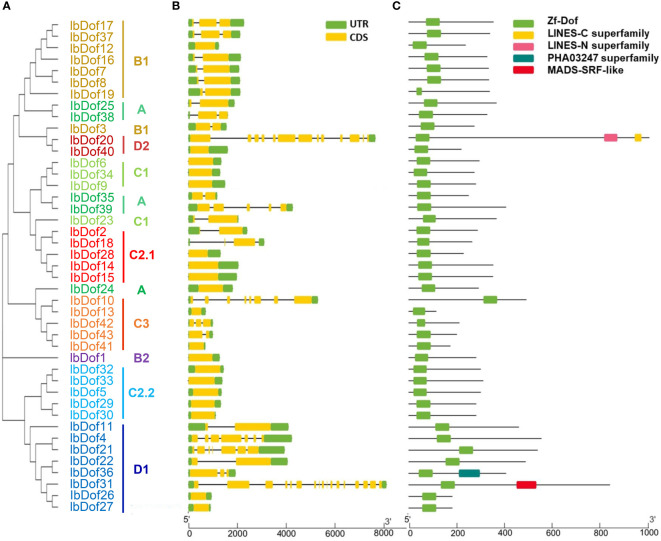
Phylogenetic relationships, gene structures and conserved domain distributions of 43 IbDof TFs in sweetpotato. **(A)** The phylogenetic tree of 43 IbDofs was constructed by MEGA X based on the consistent parameters used in Figure 2. **(B)** Gene structures of 43 *IbDof* genes. Exons and UTR are marked using yellow and green bars, respectively, black lines indicate introns. **(C)** Distributions of conserved domains detected by CD-search in the IbDof members. The colorful boxes present different conserved domains. The length of IbDofs can be estimated by the scale at the bottom.

The sequence diversities of sweetpotato IbDofs were assessed through the study of exon/intron compositions and conserved domains. In line with expectations, results from Batch CD-Search revealed that all 43 IbDof proteins possess a highly conserved Dof domain (**Figure 3**). Besides, some of the IbDofs have the other conserved parts. For insurance, IbDof20 has two additional domains, including LINES-N superfamily and LINES-C superfamily. IbDof31 and IbDof36 comprise additional PHA03247 superfamily and MADS-SRF-like domains, respectively. Notably, the number of amino acids in the Dof domain of most IbDof members is between 50 and 60. In a similar vein, our findings demonstrated that individuals belonging to the same subgroups typically have identical gene structures, even sharing similar exon/intron lengths (**Figure 3**). For example, most members of the B1 subgroup had only one intron, while multiple members from C3, D1, and D2 subgroups had the largest number of introns.

**Figure 4:**
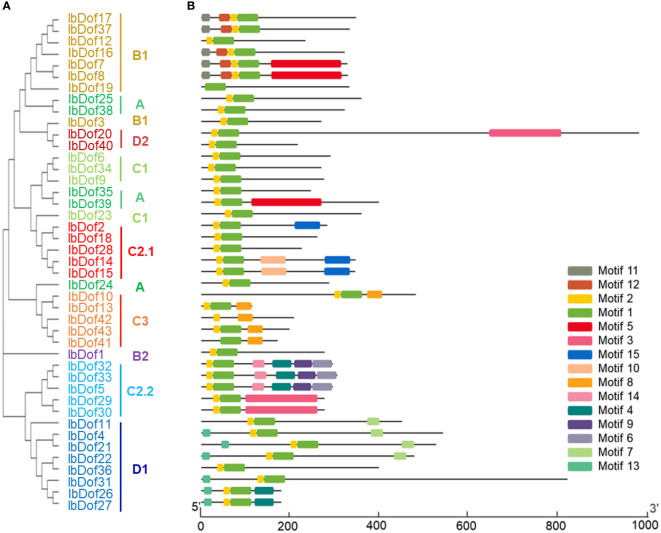
Phylogenetic relationships and conserved motif compositions in sweetpotato IbDofs. **(A)** The phylogenetic tree of 43 IbDofs was constructed by MEGA X based on the consistent parameters used in Figure 2. **(B)** Conserved motif compositions detected by MEME analysis within sweetpotato IbDofs. Boxes of different colors present different motifs, and the yellow and green boxes contained in IbDofs represent the Dof domain. The length of IbDofs can be estimated by the scale at the bottom.

In order to better analyze the sequence diversifications of IbDofs, the compositions of conserved motif were further examined. A total of 15 different conserved motifs were identified, with the N- or C-termini containing more motifs. The IbDofs within the same subgroups have comparable motif compositions, supporting the subgroup categorization, and three IbDofs (IbDof5, IbDof32, and IbDof33) belonging to the C2.2 subfamily have the most motifs (**Figure 4**). Motif 1 and motif 2 represented the conserved basic Dof domain found in practically all IbDofs, except for IbDof19 that only included motif 1.

### Collinearity studies of Dof genes between sweetpotato and other plants

**Figure 5:**
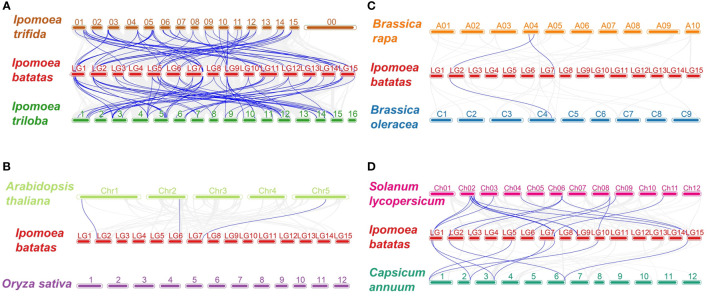
Synteny analyses of Dof genes between sweetpotato and eight representative plant species. **(A)** *Ipomoea trifida* and *Ipomoea triloba*, **(B)** *Arabidopsis thalianaa* and *Oryza sativa*, **(C)** *Brassica rapa* and *Brassica oleracea*, **(D)** *Solanum lycopersicum* and *Capsicum annuum*. The chromosomes of different plants are distinguished with differential colors. The blue lines connecting two different chromosomes highlight the syntenic Dof gene pairs within sweetpotato and other plant genomes.

To further infer the origin and evolutionary mechanisms of sweetpotato *IbDof* genes, the comparative syntenic relationships between 43 *IbDofs* and the related genes from eight representative species were investigated. These species included two likely diploid wild relative of sweetpotato (*Ipomoea triloba* and *Ipomoea trifida*), two most representative model plants (*Arabidopsis thaliana* and *Oryza sativa*), two Solanaceae plants (*Solanum lycopersicum* and *Capsicum annuum*), two Brassica plants (*Brassica rapa* and *Brassica oleracea*). 39 *IbDof* genes (90.7%) showed syntenic connections with *Ipomoea trfida* and *Ipomoea triloba*, followed by *Solanum lycopersicum* (18), *Capsicum annuum* (8), *Arabidopsis thaliana* (3), *Brassica rapa* (2), and *Brassica oleracea* (1). However, the cereal plant *Oryza sativa* did not share any such orthologous genes with the sweetpotato *IbDofs* (**Figure 5**). Notably, the collinearity between *IbDofs* and genes from *Ipomoea triloba* and *Ipomoea trifida* was significantly stronger than that found in the other six species, which may be connected to the fact that both *Ipomoea triloba* and *Ipomoea trifida* are the likely diploid wild relatives of sweetpotato.

Additionally, we discovered that 23 genes from *Ipomoea triloba* and 22 genes from *Ipomoea trifida* had a collinearity connection with two or more sweetpotato *IbDof* genes. Examples of such genes are itf01g25840.t1-*IbDof-11/-21/-31*, itf04g29810.t1-*IbDof-7/-8/-16/-17*, itb12g09820.t1-*IbDof-8/-12/-16/-17*, and itb04g29250.t1-*IbDof-7/-8/-12/-16/-17*. Besides, some sweetpotato *IbDof* genes exhibits interesting collinearity with multiple detected species, for instance, *IbDof8* was found to be collinear with three transcripts, *Arabidopsis thaliana* AT1G07640.3, *Brassica rapa* Bra035667.1, and *Brassica oleracea* Bo4g166830.1, indicating that they might be derived from a common ancestor (**Supplementary Table S7**).

### Transcriptome-wide identification of salt-responsive *IbDof* genes and their expression profiles under abiotic stress and hormone treatments

It has been found that many Dof TFs regulated different abiotic stressors (Waqas et al., 2020). In order to explore whether the *IbDof* genes identified in sweetpotato responded to abiotic stress, our previous transcriptome data were used to examine their expression levels under salt stress treatment in salt-tolerant and salt-sensitive sweetpotato cultivars. We found that a few *IbDof* genes were genotype-specific or salt stress-responsive. Then five differentially expressed *IbDof* genes, including *IbDof2*, *IbDof11*, *IbDof16*, *IbDof22*, and *IbDof36*, were selected to verify their expression profiles under various abiotic stress and hormone treatments. Taking into account the biological significance, a two-fold cut-off value of the expression difference was adopted (Zhu et al., 2015; Zhang et al., 2022).

**Figure 6:**
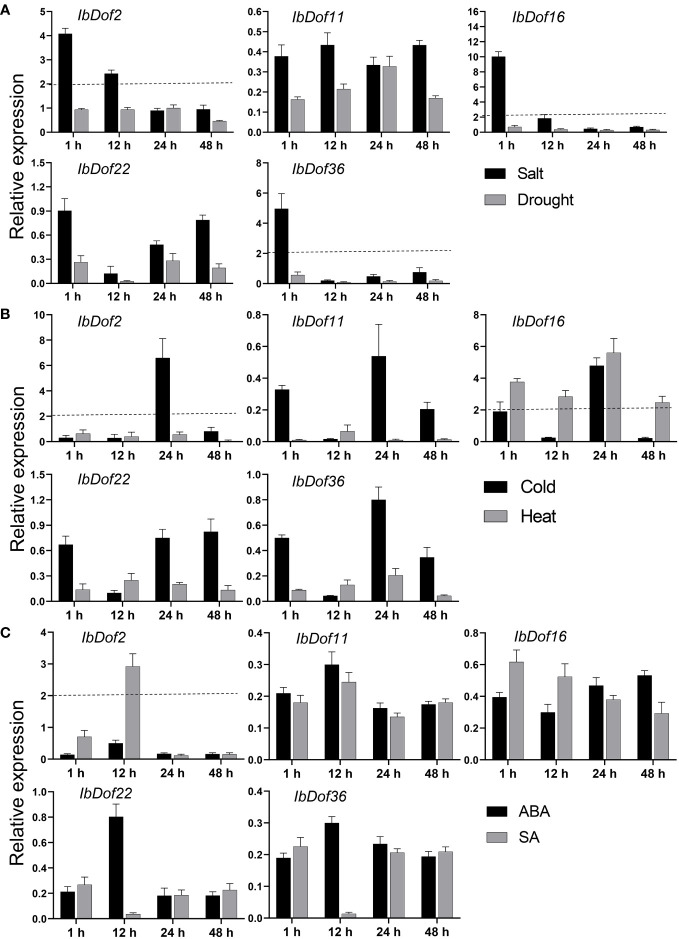
Relative expression levels of five *IbDof* genes in response to abiotic stress and hormone treatments detected by qRT-PCR. The abiotic stress treatments include salt (150 mM NaCl), drought (20% PEG6000) **(A)**, cold (4°C) and heat (42°C) **(B)**, and ABA (0.1 mM) and SA (2 mM) **(C)**. Samples were collected at 1, 12, 24, and 48 h after each treatment, the expression levels at 0 h were normalized to 1, and the Y-axis delineates the fold changes of relative expression comparing with 0 h. Bars represent the mean of three biological replicates ± SE. The two-fold threshold is presented by a dotted line.

Overall, the transcription level of *IbDof-2*/*-16*/*-36* was increased by more than four times under salt stress, and the expression of *IbDof16* had the most obvious enhancement with about 10-fold changes, while their expression was downregulated to varying degrees by drought stress (**Figure 6A**). For cold and heat stress, the mRNA level of *IbDof2* and *IbDof16* could be upregulated with 4.8-6.7-fold after cold stress, and only the transcription of *IbDof16* was enhanced with 5.7-fold after heat stress (**Figure 6B**). In addition, the transcription level of *IbDof2* was also significantly induced after SA treatment, about 2.7 times that of the control (**Figure 6C**). Therefore, sweetpotato *IbDof* genes may act as crucial and distinct regulators in response to abiotic stress, according to their considerable and varied transcriptions.

### Cis-element prediction in the promoters of *IbDof* genes

**Figure 7:**
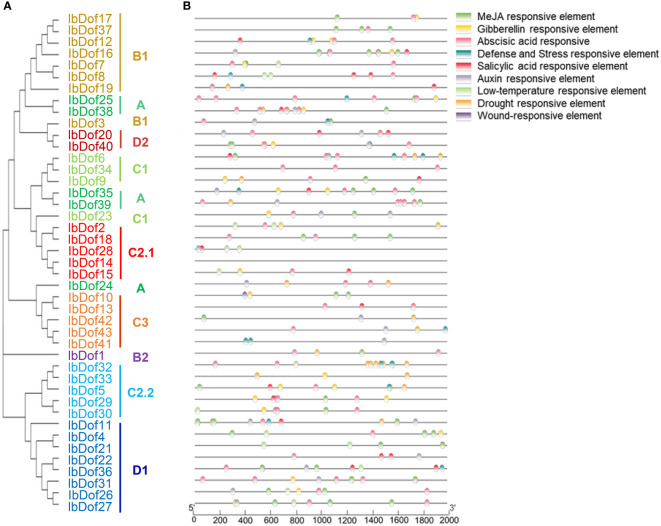
Phylogenetic clustering and predicted stress- and hormone-related cis-elements in the promoters of *IbDof* genes. **(A)** The phylogenetic tree of 43 IbDofs was constructed by MEGA X based on the consistent parameters used in Figure 2. **(B)** Predicted cis-elements in the *IbDof* promoters. 2000 bp promoter regions of each *IbDof* gene were detected by PlantCARE database. Different colored rectangles represent different cis-elements that are potentially involved in stress or hormone regulation.

In order to comprehend the potential regulatory mechanism of *IbDof* genes in response to abiotic stress and hormone treatments, the specific cis-elements in the promoter of *IbDof* genes were analyzed (**Figure 7**). The results showed that 97% of the promoters featured numerous regulatory elements sensitive to stresses, including the drought responsive element (MBS), the defense and stress response element (TC-rich repeats), the low temperature responsive element (LTR), and the wound responsive element (WUN-motif) (**Supplementary Table S8**). The transcription of multiple *IbDof* genes, including *IbDof-2*/*-16*/*-36*, was significantly induced by different abiotic stresses or hormones. Accordingly, their promoters were enriched with stress-related cis-acting elements such as MBS, TC-rich repeats or LTR.

Besides, all *IbDof* promoters contained various hormone-related cis-elements, including the GA response element (GARE-motif, P-box, TATC-box), the MeJA response element (TGCCG-, TGACG- and CGTCA-motif), the auxin response element (TGA-box, AuxRR-core), and the ABA response element (ABRE). Approximately 84% of the promoters have an ABA response element. The information indicates that these cis-elements have a potential role in affecting the response of sweetpotato plants to abiotic stresses.

### Interaction network analysis of the IbDof proteins in sweetpotato

**Figure 8:**
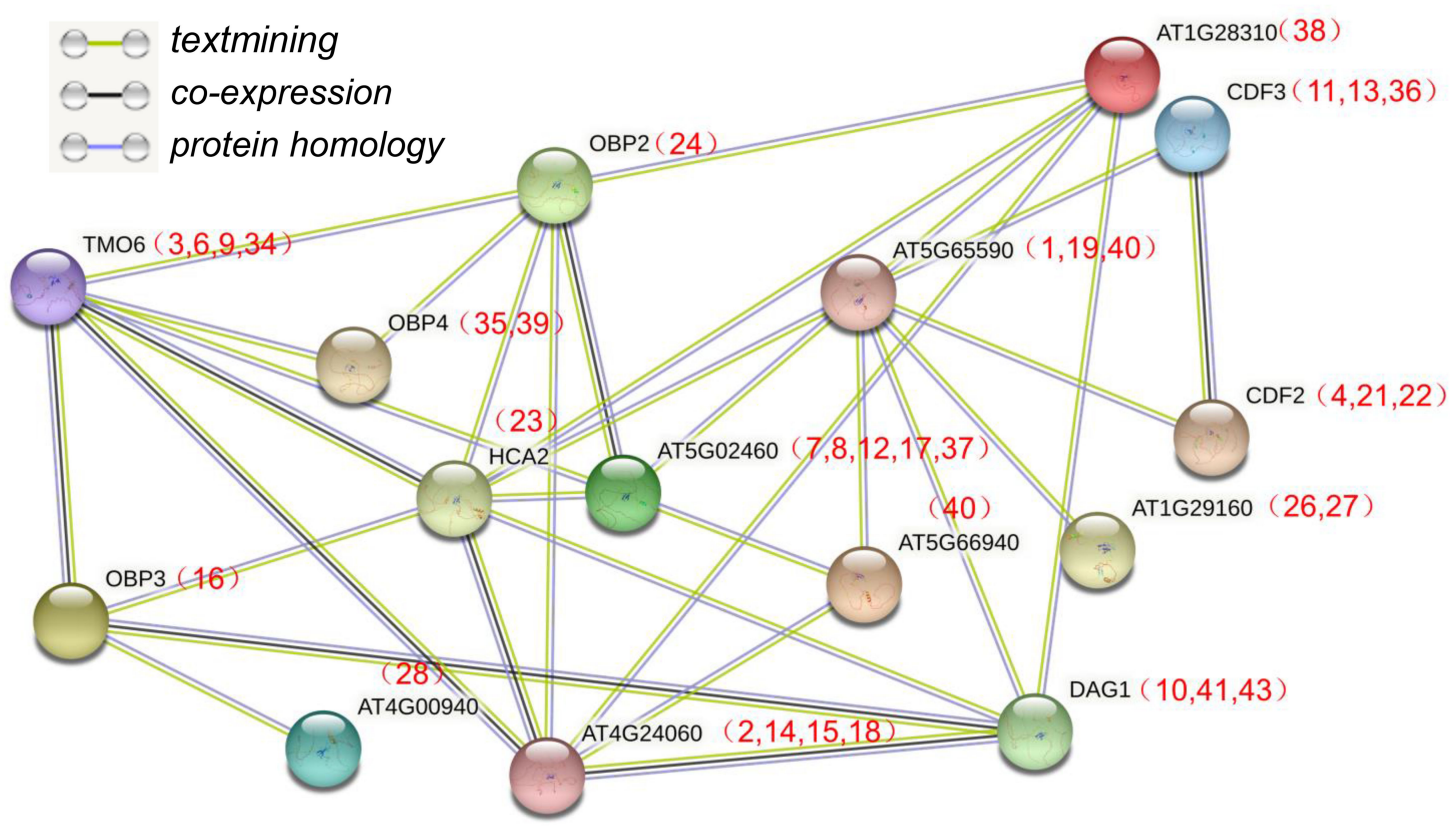
Interaction networks of IbDof proteins in sweetpotato according to the orthologues in Arabidopsis. The amino acid sequences of IbDofs were employed to search the STRING database, network node represents proteins, and edge represents protein–protein associations. The colored lines between the nodes indicate the different kinds of interactions. The numbers (*IbDof* gene number) in brackets represent the corresponding orthologues in sweetpotato.

The Dof domain in Dof TFs is a critical domain that can mediate protein-protein interactions (Waqas et al., 2020), indicating that IbDof TFs may also function by forming protein complexes. In order to further study the potential interaction between IbDofs, an interaction network associated with sweetpotato IbDofs was constructed based on the orthologs of Arabidopsis AtDofs by the STRING database (**Figure 8**). The results indicated the prospect of protein interaction between sweetpotato homologous IbDofs corresponding to the Arabidopsis AtDofs, such as TMO6 (IbDof-3/-6/-9/-24) and OBP2 (IbDof24)/OBP3 (IbDof16)/OBP4 (IbDof35 and IbDof39)/HCA2 (IbDof23). The sweetpotato IbDof interaction network indicates a potential complex association, suggesting a possible way for IbDof TFs in regulating the response of sweetpotato to environmental stresses.

### Detection of transactivation activity and protein interaction of selected IbDofs

**Figure 9:**
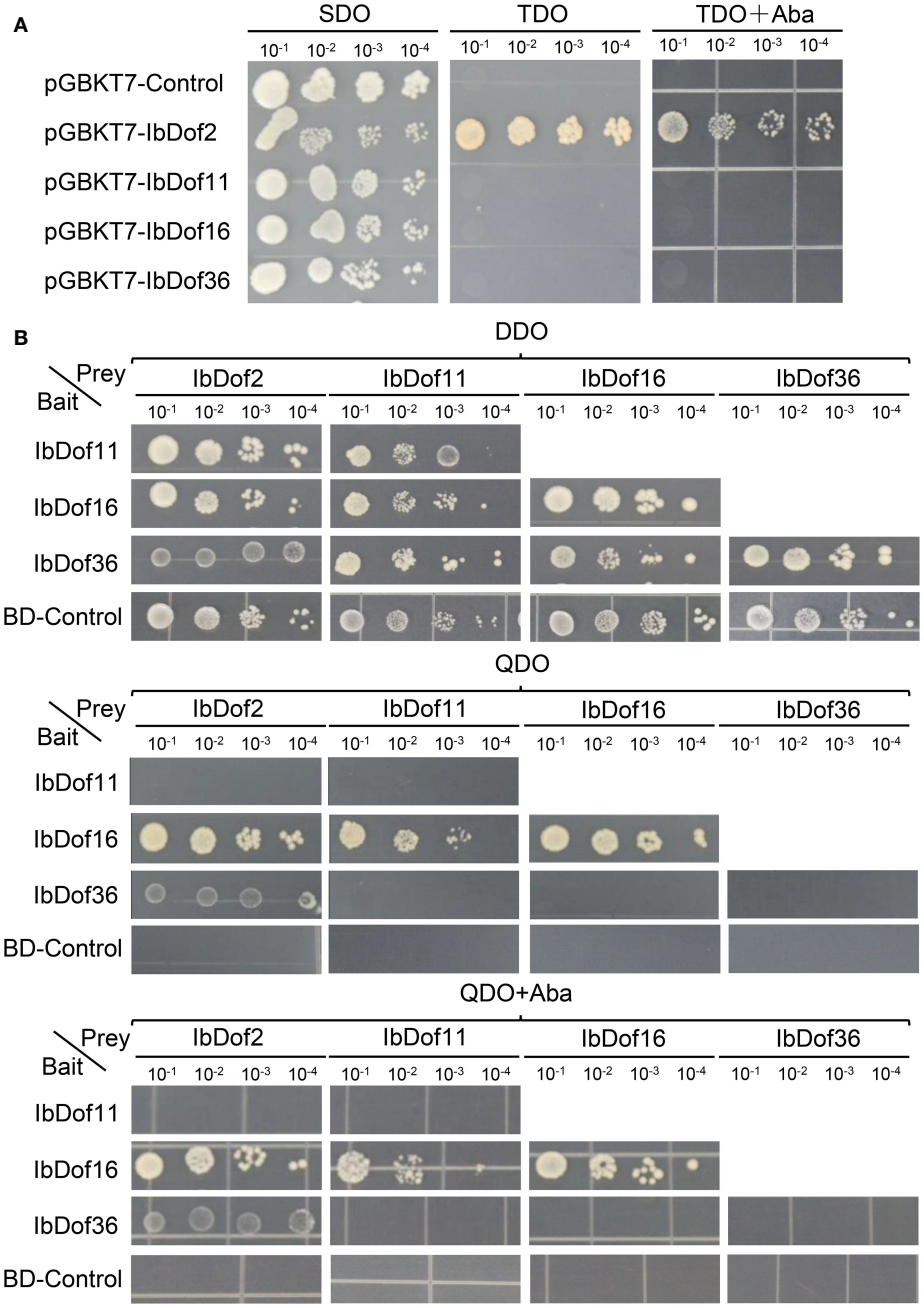
Analysis of transactivation activities and protein interactions of IbDof proteins. **(A)** Transactivation assays of IbDof-2/-11/-16/-36 in yeast strain Y2Hgold. Yeasts containing pGBKT7-IbDof-2/-11/-16/-36 or pGBKT7 empty vector (negative control) were streaked on the SDO (SD medium lacking Trp); TDO (SD medium lacking Trp, His, Ade) and TDO medium with 200 ng/mL AbA, respectively. **(B)** Yeasts containing both the indicated recombined pGBKT7 and pGADT7 plasmids were streaked on DDO (SD/-Trp-Leu) medium, QDO (SD/-Trp-Leu-His-Ade) medium with or without 200 ng/mL AbA. All the plates were recorded three days after 30° of incubation.

Considering that the transcription levels of *IbDof-2/-11/-16/-36* genes were upregulated by different abiotic stresses or hormones, they were chosen to detect possible transactivation activities in yeasts by constructing the recombinant pGBKT7 vectors. The results displayed that all the yeasts can grow normally on SDO medium. However, only the recombinant pGBKT7-*IbDof2* yeasts can survive on the TDO medium with or without AbA, while the control pGBKT7 vector and the recombinant pGBKT7-*IbDof-11/-16/-36* can not (**Figure 9A**). The results showed that IbDof2 TF has transactivation activity in yeasts, while IbDof-11/-16/-36 does not.

Besides, the possible interactions between any two of IbDof-2/-11/-16/-36 proteins (including the interaction with themselves) were further detected by Y2H assays, but pGBKT7-IbDof2 vector was not involved because of its self-activation activity. The results presented that the yeast transformed with both the recombinant pGADT7 vector and the recombinant or empty pGBKT7 vector could grow normally on the control DDO medium. And the results showed that IbDof16 could interact with itself in addition to IbDof2 and IbDof11, and IbDof2 could also interact with IbDof36. Other than that, no visible interaction was detected in other combinations (**Figure 9B**)

## Discussion

Dof proteins represent one of the plant-specific TF families and play a substantial regulatory role in the resistance mechanism against various abiotic stresses in plants (Noguero et al., 2013; Waqas et al., 2020). Sweetpotato is not only a crucial food crop, but also widely used in animal feed and industrial materials. In addition, sweetpotato has a variety of natural advantages that other common crops do not have, such as wide adaptability, high yield, and strong stress resistance (Liu, 2017). However, the specifi;c roles of most Dof genes remain unknown, and the Dof TFs in sweetpotato has not been comprehensively characterized so far. The development of bioinformatics and the completion of sweetpotato genome sequencing provide a strong basis for genome-wide identification of certain gene families (Yang et al., 2017b). In this work, the Dof TFs in sweetpotato were systematically investigated, and the identification and characteristic of stress-related *IbDofs* provide worthy information for the further functional exploration of *IbDof* genes in stress tolerance.

### Diverse characterizations of IbDof TFs in sweetpotato

In this study, a total of 43 *IbDof* genes were identified in the sweetpotato genomes. These genes were unevenly distributed across the chromosomes and did not correspond to chromosome size, which may be caused by the unequal gene duplications of chromosomal segments. No *IbDof* gene was observed on Chr4, probably because of fragment loss or chromosome translocation during the evolution (Zhang et al., 2022). Comparable distributions were also observed in sorghum (Xiao et al., 2022), cabbage (Ma et al., 2015) and wheat (Fang et al., 2020), demonstrating that the number of Dof members was not closely related to the size of chromosomes. Although each IbDof TF has a conserved Dof domain, there are still considerable variances in the molecular features of IbDofs, which may be due to changes in their non-conserved regions. The significant variability indicates high complexity of sweetpotato IbDof TFs. Additionally, the number of 43 *IbDof* genes is more than that in *Arabidopsis* (36) (Lijavetzky et al., 2003), rice (30) (Khan et al., 2021), tomato (34) (Cai et al., 2013), pepper (33) (Wu et al., 2016), sorghum (30) (Xiao et al., 2022), and potato (35) (Venkatesh and Park, 2015), but much lower than that in *Saccharum officinarum* (119) (Gupta et al., 2014), *Gossypium hirsutum* (114) (Li et al., 2018), *Brassica napus* (117) (Lohani et al., 2021), sugarcane (119 members) (Gupta et al., 2014), and wheat (108 members) (Fang et al., 2020), indicating that the amount of Dof TFs varies greatly in monocotyledons and dicotyledons.

Phylogenetic analysis showed that 43 IbDofs were classified into nine subgroups based on sequence homologies from known Dof family members from Arabidopsis and rice (Lijavetzky et al., 2003), consistent with the results of the Dof TFs in multiple plants such as rose (Nan et al., 2021), watermelon (Zhou et al., 2020), Tartary buckwheat (Li et al., 2022), *Gossypium hirsutum* (114) (Li et al., 2018), and *Brassica napus* (117) (Lohani et al., 2021). The gene structures can be applied as supporting evidence to confirm the evolutionary relationships among genes or organisms (Koralewski and Krutovsky, 2011). Gene structure analysis showed that there were significant differences amongst different subgroups, while similar structures were detected within the same subgroup, as was the case in motif analysis. For example, multiple subgroups contain their own unique motifs. The findings suggest the potential functional differentiation of IbDofs among different subgroups. And the intron numbers of *IbDof* genes were relatively small, most genes had only one intron or even no intron, which was similar to the Dof genes in wheat (Fang et al., 2020), rose (Nan et al., 2021), and Tartary buckwheat (Li et al., 2022). As reported in the previous result, the intron-less genes may be associated with the quick stress response (Zhang et al., 2022).

Many plant gene families have evolved and expanded as a result of gene replication events, which may also encourage the formation of new functional genes and species that can better withstand harsh environments as plants evolve (Chen and Cao, 2015). Previous collinear studies on Dof gene family in Tartary buckwheat (Li et al., 2022), rose (Nan et al., 2021), and *Gossypium hirsutum* (Li et al., 2018) showed that segmental duplication events played a dominant role in Dof gene expansion. Similarly, no tandem repeat events were observed among sweetpotato *IbDof* genes, and segmental duplications were found to have a primary contribution to their expansions, indicating some *IbDof* genes may have originated from gene duplications in sweetpotato. However, studies also displayed that both tandem and segmental duplications exist simultaneously in the Dofs from *Brassica napus* (Lohani et al., 2021), wheat (Fang et al., 2020), and poplar (Wang et al., 2017).

Collinearity analysis can provide valuable insights into the evolutionary history of species (Wang et al., 2012). The synteny study examining the connections between *IbDof* genes and the equivalents from eight representative plant species were investigated. The biggest number of orthologous genes among them were found between sweetpotato and *Ipomoea triloba* and *Ipomoea trifida*, which was supported by their close evolutionary relationships, followed by tomato, pepper and Arabidopsis. These orthologous pairings may share common ancestors with the related plants and sweetpotato. In addition, a more intricate link between a single *Ipomoea triloba*/*Ipomoea trifida* gene and multiple sweetpotato *IbDof* genes was detected, suggesting that these orthologous genes may play an important role in the evolution of sweetpotato *IbDofs*. No orthologous gene pair was observed between sweetpotato and rice, probably due to numerous chromosomal rearrangements or fusions in the genomes (Paterson et al., 2012).

### Expression profiling and functional prediction of *IbDofs* in sweetpotato

An increasing number of studies on the crucial roles of Dof TFs in regulating plant responses to multiple harmful environmental stimuli suggest that Dofs are excellent candidates for improving agricultural stress resistance through molecular breeding (Waqas et al., 2020). For instance, overexpression of tomato cycling DOF factor SlCDF1/SlCDF3 or grain amaranth AhDOF TF in Arabidopsis all showed enhanced drought and salt tolerance (Corrales et al., 2014; Massange-Sanchez et al., 2016). Besides, *Juglans regia* JrDof3 contributed to enhance the high temperature stress response of JrGRAS2, which could significantly regulate the expression of *HSPs* to improve high temperature stress tolerance (Yang et al., 2018). However, our understanding of how Dof TFs regulate stress responses in most plants, including sweetpotato, is still quite limited. The cis-elements present in the promoter regions play a key role in gene expression regulation (Meng et al., 2023). Numerous hormone- and stress-related elements in the promoters of *IbDof* genes were detected, such as the MBS, TC-rich repeats, P-box, and ABRE. Our transcriptome data and qRT-PCR results also revealed that the expression of multiple *IbDof* genes was clearly differed in response to different abiotic stress and hormone treatments, suggesting that sweetpotato *IbDof* genes may also have important and varied roles in response to environmental stresses. The data are supported by previous similar results, as many *TaDof* genes in wheat and *RchDof* genes in rose were also obviously upregulated by salt and drought treatments (Fang et al., 2020). And the transcription levels of Arabidopsis *CDF3* were significantly induced by drought, extreme temperature and ABA treatments, the transgenic assays showed that overexpression of *CDF3* could significantly improve the drought, cold and osmotic stress tolerance (Corrales et al., 2017; Nan et al., 2021). Therefore, sweetpotato *IbDof2*, *IbDof16*, and *IbDof36* genes showed obviously increased expression after stress treatments, indicating the accumulations of these genes may lessen the harm caused by unfavorable stresses in our analysis, but additional experimental confirmation is needed.

The dimerization of Dof TFs with other proteins depends on the conserved Dof domain (Gupta et al., 2015). The first protein-protein interaction of Dof TFs was found in Arabidopsis in which AtOBP1 interacted with the bZIP protein to regulate stress response. Similarly, many Dof proteins have also been found to exert their regulatory functions by forming dimers such as OsDof3 by interacting with GAMYB in rice, Dof1 by interacting with Dof2 and HMG1 in maize, and Dof 3.2 by interacting with TCP14 in Arabidopsis (Yanagisawa, 1997; Waqas et al., 2020). In the present study, the STRING database predicted that sweetpotato IbDof TFs may participate in the stress tolerance through a complicated protein interaction network based on the homologous proteins of Arabidopsis. For example, TMO6 (IbDof-3/-6/-9/-24) might interact with numerous Dof proteins including OBP2 (IbDof24), OBP3 (IbDof16), OBP4 (IbDof35 and IbDof39), and HCA2 (IbDof23). And IbDof16 could interact with IbDof2, IbDof11 and itself, and IbDof2 could also interact with IbDof36 according to the subsequent Y2H studies, indicating a complicated interaction connection amongst sweetpotato IbDof proteins. Collectively, our findings imply that multiple stress-responsive IbDof TFs can form intricate complexes with Dof and other types of proteins through direct protein-protein interactions, which may exert crucial roles in abiotic stress signaling cascades.

Collectively, in this study, we found that 43 *IbDof* genes were unevenly distributed on 14 of the 15 chromosomes of cultivated sweetpotato. The majority of *IbDof* genes lacked introns, and these IbDofs could be divided into nine subgroups according to the phylogenetic analysis. The *IbDof* genes within the same subgroup generally shared similar gene structures and motif compositions, while they were distinguishable among different subgroups. Segmental duplication events were shown to be the prominent driving force for the expansion of sweetpotato *IbDof* genes, and a collinearity analysis of orthologous genes from eight typical plants gave important hints about the evolutionary traits of the *IbDof* genes. RNA-seq data and qRT-PCR detection showed that multiple *IbDof* genes, particularly the *IbDof2*, *IbDof16* and *IbDof36*, were significantly upregulated in response to abiotic stressors and hormones, indicating that they might play a pivotal role in stress resistance. Additionally, IbDof2 protein has obvious transactivation activity, and a complicated interaction relationship between IbDof TFs was found, suggesting the complex connection and regulatory mechanism for IbDof TFs in regulating the response of sweetpotato to environmental stresses. These findings provide valuable information for further comprehending the intricacy and importance of the Dof gene family, and multiple IbDof members with promising prospects in regulating sweetpotato response to environmental stresses are expected.

## Data availability statement

The datasets presented in this study can be found in online repositories. The names of the repository/repositories and accession number(s) can be found in the article/**Supplementary Material**.

## Author contributions

XM and MZ designed the experiments, CZ, MZ, and XM analyzed the data and wrote the manuscript. CZ, TD, JY, HH, SL, FG, and HM performed the experiments and analyzed the data, TD and JZ improved the manuscript. All authors contributed to the article and approved the submitted version.
