# Supplementary material for: Genome-wide survey and expression analysis of Dof transcription factor family in sweetpotato shed light on their promising functions in stress tolerance
Source: Front Plant Sci. 2023 Feb 21;14:1140727. doi: 10.3389/fpls.2023.1140727 (PMC9989284; doi:10.3389/fpls.2023.1140727)

**Supplementary Figure S1**. Chromosomal map showing the uneven distribution of 43 *IbDof* genes on 15 sweetpotato chromosomes except Chr4. The chromosome numbers are indicated to the left of each chromosome as LG1-LG15. The scales were indicated the genome size of sweetpotato genome (Mb).


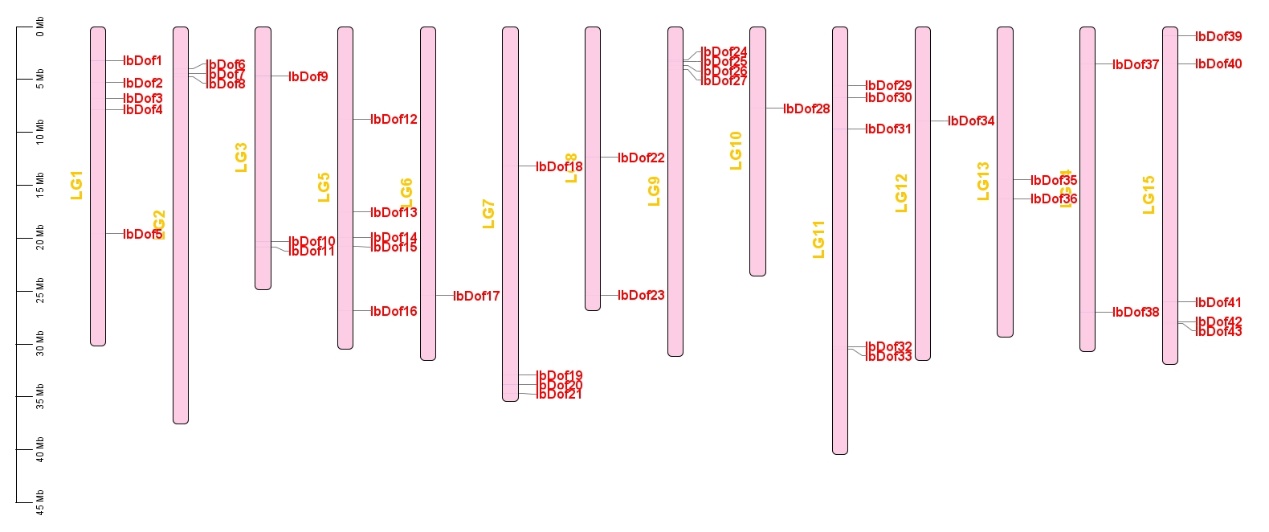

Supplement: Supplementary file 1 [file DataSheet_1.docx]
