# Supplementary material for: Genome-wide survey and expression analysis of Dof transcription factor family in sweetpotato shed light on their promising functions in stress tolerance
Source: Front Plant Sci. 2023 Feb 21;14:1140727. doi: 10.3389/fpls.2023.1140727 (PMC9989284; doi:10.3389/fpls.2023.1140727)
Supplement: Supplementary file 3 [file Table_2.docx]

## Supplementary Table S2. The nucleotide and amino acid sequences of 43 IbDofs identified in sweetpotato genomes.

**Nucleotide sequences**

>IbDof1

ATGGCAGCAGCCAACAAAGGTGATGACCAGATGTCCGGCGACGGCGACAAAACTCCGGCGGTGACACGGCGGCTGAAATGCCCAAGATGTGATTCGCCAGACACCAAGTTTTGCTACTACAATAACTACAGCCAATCCCAACCAAGGTACTTCTGCAAGACGTGCAGAAGGTACTGGACTAAAGGAGGCGCGTTGCGCACAATCCCCGTCGGCGGCGGCTGCCGGAGAAGAAAAATCATGAAATCTTCCCCCGCCGCAAGATATTATGATATCGGCGGATTGGGATACCTTAACGGTGTCTCTCAACCCTTGGGTGGGATAAAAATCCCCAGTTACAACACTTCAGCCGCCGCTCTTATCGACCAATTATCCGGCGCTCTTTTGGCTATGCCGCAAAACCCGCCTTGCTTCAATCTCGACCAATTATCGCCGCCGTTCTCCATTTCCAGTCCTTTATTTGACTTCAAATTTCCTGGAACTCAAGAACTGGGCAGGCTTGCACCCCCAATCGAGCCGGTGGGCACTGTAAATCAAGATTTGCAGTGGAATTTACAGCAGAAAATTCTTGAGAAACAGATTCTTGAAATTCCGACGCCACCATCTGAAGCCACTTTTGAAAGCAGTAGTTCAAGAATCGCTGAAGCTACTGAATGGCTCTTTGACGACGACGATGTTACTTTTCCACTCCCTGCAAACCAAACTGCAACAAATAGTTTTGGAAATGGGAATGGAAACGCCTCAGAAGACGACTGGGACGAACTCATTCAAGCTTGGGCCACAAACTTCAATGAATTCAGTGCTTTGCCATAG

>IbDof2

ATGGATTCTTCTCAGTGGCCACAGGGAATAGGGGTAGTGAAGGGTGGTGTTGTGGAGGCTCCAAAGAAGCCAAGGCCGGAAAAGGAAAAAGCTTTGAATTGTCCGAGGTGCAGTTCCACCAACACAAAGTTCTGTTACTACAACAATTATAGTCTGTCGCAGCCGAGGTACTTTTGTAAGACATGTCGAAGGTACTGGACAGAAGGGGGTTCTTTGAGGAATGTTCCAGTGGGGGGAGGCTCAAGAAAGAACAGAAGATCATCATCATCCATTTCTTCTTCTTCATCTCTAGCTTCTTCTTCATCTTCTTCCAAGAAACTTCCAGATCTTGCTGTGACTCCCCCGAGTTTTCCCTCTCATCAGCAAAACCCTAAAGGGCATGATCTCAACCTGGCTTACCCGCCGCCGCAAATCAATAATTTCGGCGGCGCCGGTGGGATTTCGGAGATTCTTAATCTGAACCCTAATTCCTCCGCCGCCATGGAGATTCTCAAGAATGGGTTTTCTTCGTTCATGGGCATGCCGCCTCCGGTGCATCCAGATCATCATCATCATCATTCCAACGCCGCCATGTTCTTGTCTCCCGGAGTTCCCTTGTCATTGGATGGGTTCTGTAATGGGTATGGGAATCTCCCGCCGCAACCACAACCTGCGGGGACAGCTAGGATGTTTTTCCCTAATCTTGAGGACTTGAAACCTGTCTCAACTATGGCGACTGATAATCATCAGTTTGAGGAGCAGAATAGAGGGGAAGGGGAGAATGATGGGAGTGGGTATTGGAATGGAATGCTTGGGGAGGAGGAGGAGGAGGAACATCATGGTAAATAA

>IbDof3

ATGATTCAGGAGCTTTTAGCAGCTGGGAATGGTGGGCTGCTTGGAGGAGGGGAAAGTAATAATAATGCTCCAAAGCTATCCATTACAGATAATTCGTCTGGTCCTTTGGTTTCGTCTTCTCCGACGTCGAGGTCGGAGAATTTAAGGTGCCCGCGCTGCGATTCTTCTAACACGAAGTTCTGTTACTATAATAACTATAATCTCACGCAGCCGCGCCATTTCTGCAAGACTTGCCGCCGTTACTGGACAAAAGGAGGCGCGTTGCGTAACGTCCCCATCGGAGGCGGCTGCCGGAAAAACAAGAACGCCATCGTGAGCGCGTCTATGGGGAAATCCGCCGCCGCCGCGAGTACGAAGTGGAAGTCTAGTTTTCTTGGCGGATTGGAACCGGAGATTCCATCGTCGAAAAGTTTCCTGTTTGGACCGCCCCAGAACACTAATCATCAGTACCCCTTTCTTTCCCTATTAAGATCCGGTCAAAACCCTAATCCCGGCGCGAACGGATTCATTCTCGCCGGCGGCGAAGGTCAGGCCGTGGGAATGCAAGAGCTGATATATCAAAGACTCATGAAATCTTCATCATCGTCGTCCGCAACGAACAACTACAACGTTAATTGCTACGAGCACCCGCTAGCGCTCCTCGGCAATGCGGCTTCATCCTCGTCCTCCGTTTTGTCGCCGGCGATTTTGGAGTCTGCTCCGGTGTCGGCGGCCGGAGAATTCGGTTACTGGAACCCTTCACTTTCCACGTGGTCCGACCTGCCGACAACCGGCGGCGCATATCCGTGA

>IbDof4

ATGCCAGAAGTGAGCAAAGACCCCGCGATAAAGCTGTTTGGGAAGACTATCCAGTTGCCGGATGCTCCGGCGCCGGCTCCGGCGACGGAGATGGATGTCGATCCCGGCGGGGGAGCAGCTTCATCTCCTCCTCCTGCTGAGGAATTCTCAGCTAAAGATAGTTCTTGTTCAGAAGCTGAGGGCGGAGAGGATGAACAACTGCAAAAGAATGAAAATGGAGAAAGTGAAGAGGATGAAGGAACAGTGTTATTGGAAGGGGAAGAGATAATGGATGAGAGTGTAGATGAAATGAGGGATGGGTTAAAAGCACCACCTGTTGATGAAGATGACAAGATAATTCCATGTCCTAGGTGTGATAGTATGGAGACCAAGTTCTGTTATTTCAACAACTACAACGTGAACCAGCCGCGCCACTTCTGCAAGAAATGCCAGAGATATTGGACGTCGGGTGGGATGACGAGGAATTTGCCTGTTGGCGCTGGGCGCAGAAAAAGTAAGAACTCGGCTGCTCATTATCACCATTTGAATGCTGTGAAGTCGTTTCAGAGTGCTCGTGTAGACCATCCCGAGAGAATCCAACACCATGCTCTTAAGGCCAATGGAAAGGTGCTTACGAATGGCTCGCTAAATGGGTTTTACAAGCCAGTAGAGTTAGGAATCCCGGTTTCATATGGAGCTGGGGATAATGGAGCTGACCATTCAAGTGGTTCCTCATCATCATCAAGTACAAAGGATGATGTTTGCAAAAATGGGTTGCCCGACATGATGAGACAGAACTACCATGGCTTCGCTCCTCAATTACCGTGCTTTCCAGGTCCTCCTTGGCCATATCCATCCAGTGCTGTTCAGTGGAGCTCTGCAGTTCCTCCACCTGGTTGCTTTTCTCCCGGCTTTCCCATGCCATTTTACCCCCCGGCAATGTATTTGGGTTATACCATTCCATGCTCTTGGAATGCGCCTTGGGTGAACAGACCTGTTTCGCCCCAACAGCACACGTCTTCTGGCCCTAATTCTCCAACCTTAGGGAAGCACTCAAGGGATGACAACGCGGTGACACCAGCCAGCATCAAGGAAGGGGAGCCACCCAAAGATAGTAATCCAGAGAAGTGTCTGTGGGTTCCTAAAACTTTGCGGTTTGATGATCCAGAAGACGCTGCAAAGAGTTCCATATGGGCAACGCTGGGAATAAAACACGATAGAGTTGATTCGATTAGGGGTGGCGCTTTTAAGGCCTTCAAGTTGAAGGATGACGAGAAGAGCCATGCTTCAAAGAACTGTAGAGTATTAGAAGCCAATCCAGCTGCATTATCCAGCGAGCAACCTTGGGTTATGACTTTACGATCATATAATATTTTCTCGAAGATCAGACAACTAGATGCGGATTTCCAGCTTTTTGTTCTGAGAAAGGAGAGGTTCTATTCCATAGATGAGCAGAGGATTGGCTTGTTGGCAGCAACCAGAGGATGCCCGAGCCCCGAGGTTGGGCAGAGACGCGGAAGAGCCACAACGAAGGTAACACCGGCAATGTATAGTAGCCTAAGATGCACCTGTCATAAAGACATGTTTGATATCAAGCTAAACATTGTGTGA

>IbDof5

ATGATGGAGGGAGCGCCGGCGACATGGAAGCCTAACGTTGAGATTTCTCCGACCTGTCCTCGTTGCGGTTCCATGAACACCAAATTCTGTTACTACAACAATTACAGCTTGACCCAACCTAGGTACTTCTGCAAAGGTTGTCGGAGGTACTGGACCAAAGGGGGTTCTCTCCGCAACGTTCCCGTCGGCGGCGGCTGCCGGAAGACCCGGAGAGCCAAATCCTCGTCTATTCGCGTTGTCTCCAATCATCGCCGAGGAGGGGTGTTTGGGATGTCCGCCGCCGACAACAATAACCCCGGCGGATCCACTACTAGTCCCGCCTCCGCCGCCGCCGGGACTAATGGCCCGGCCCCCAACATTGATCTCGCCGCCGTTTATGCGAACTTCATGAATCCCAATCCTAATCCTCAACCTCAACCCGCCCAAATCCCGGAAACTACACTGCCTAATAATAATGACGGCGGCGGTGGCGAAGGTCCATCGTTTGAATTTTCCGGCTATCCAGCAATGTTGAATGTGGATTATTTCGTCCCCGAAGCCAACATGGCTCCTCAAGACGGCGGCTTCGTCGTCGACGAATTTGGAAACAATAATAATTCCGCGGCAGCGCTGTTTCAGGAACAATTCTGCGGCGAGTCGTTGCCCCCAATACTCCCTCCTCATGAAGAGTTAACAACCTCGGAAGGGTGGCCCACCCACAATTCCGACATGAATATGAATATGAATATGATGTTTCCATTGCATCACACAATAAGTGCTCATCATCATGAACCAGAGCTACAAGGTTGTCCTAACCATCACACCACTTCAAGTTCAAGTCTTTTCAGCATCCCCACCACTTATGATTCCATTTTCCGGCCTTAA

>IbDof6

ATGTCATCACAGACGCTAGAAAGCATGATGGTGGCGTCGGCAAAGGCGCAGCAGCAAGAGAAGAAGCCACGGCCGGCGGACGAGCACGCCCAGAAATGCCCTAGATGCGAATCCACCAACACCAAATTCTGTTACTACAACAACTACAGCCTCTCTCAGCCGCGCTACTTCTGCAAATCCTGCCGGAGATACTGGACCAAAGGCGGCACCCTCCGCAACGTCCCCGTCGGCGGCGGCTGCCGGAAAAACAGACGCTCCTCCTCCTCCAAAGCCAGAATCTCAACCGCCCAACAAGATCCCCATACCTCTAGCCCCGTCCTCACAAATTTCCCCTACGAATCCCCCCACGATTTAGGCCTCACATTTGCGAGCTTGCAGAAACAAGCGGACGGGAATCTAGGGTTGGAAGATCACGAAATGGCGGCGCCGATGATGTGTAATCCCAATAATACCCTCTCTGATGTTCTCGGAAACTACGGCGCCGCCTCACACGGCTTTCTTGATTCCCTAAGGGCTACCGGATTCCTCGAACCCCCAACAAACGGATTGTACCACAACCACAGCCTGCAGTACTACGGGACCGTGGGGGAAATGGGAATGCCCTACGATGTTCACATGGGTGGTGGGGTGTCCACGTCAGCTGCCGTGAAGCAAGAGATGTACAGCAATGGAAGAGATAATGAAGGTGATGATGACAACAGGGTATTATGGGGATTTCCATGGCAACATGGAGGAGGAGAAGGCAACATGAACATGGTTGTTAATGATGTTGAATCAAGCAGACAAAGCAGTTGCAATATTGGGTTTGGTTCTTCTTGGCATGGCCTTGTCAACAGCCCTCTCATGTAG

>IbDof7

ATGGTTTTCTCTTCTTTTCCTGTCTATTTAGATCATTCCAATTTGCACCAGTTACAACAGTTTGCTGCAGGTTACCATCAACAAGGAACTGGCCTCGAGAATCCTCAGATTTCGACGCTGCCGGCGCCGGTGGGTGCCGGGCAGGGATCGATCCGGCCCGGGTCGATGGTGGATCGAGCCCGGCTAGCCCGGATGCCTCAGCCGGAAAACGGTTTAAAGTGTCCCAGGTGTGATTCCACTAGTACCAAGTTTTGTTATTACAATAACTACAGCCTTTCGCAGCCTCGTCACTTTTGCAAGACGTGCCGCCGTTACTGGACTAGAGGGGGCGCTATGAGAAACGTTCCGGTGGGTGGAGGCTGCCGGAGGAACAAGAGGAGTAAGAAGTCCGGGGGAGCCGCCGCGGCTGTCTCCGACAAACAAAGCGGCGGTGGCGGCGCCGGGAATAGTAATAATATTAATACTTCCACCGCCGCCCATATTTCATGCCCTACTAGCTGCGGAAGCGCGGAAATTTCCGGCAACTTTTCACAGCCGCCACTCATGGCCGCGTTTCAAGCCGGCTTAAACCATTTCGGCGGCTTCCAGCCTCCGCAACTGGTGGCTAACGAAAACGGAGGGTTTGGTGATATGAGCTTTCAATTAGGAGCCGAGCCTTGGAGATTGCCGGCGTCTTTAACTGCCTTTGACACGCCGCCACCCACAAATCTATTCCCTTTCCAAAACGAAGCAATTGAAGCTTCATCTTCCCGCGCAACCCCCGAGTTTCCGCAGCTCACATCATCGGTAAAAATGGAAGACACCCAAGGGCTGAACAGTAATTCAACAAAGCAGTTCCTCAGCGCTCTTGGAAACAACCAATTCTGGGGTGGCGCCGGTGGCAACACTTGGACTACCGGAGGCTTTTCCGGCCATCTCAACTCCTCTTCCACTTCATCTTCCACCACCCATCTTCTATGA

>IbDof8

ATGGTTTTCTCTTCTTTTCCTGTCTATTTAGATCATTCCAATTTGCACCAGTTACAGCAGTCTGCTGCAGGTTACCATCAACAAGGAACTGGCCTCGAGAATCCTCAGATTTCGACACTGCCGGTGCCGGCGCCGGTGGGCGCCGGGCAGGGCTCGATCCGGCCTGGTTCGATGGTGGATCGAGCCCGGCTAGCCCGGATGCCTCAGCCGGAAAACGGTTTAAAGTGTCCCAGGTGTGATTCCACGAGCACCAAGTTTTGTTACTACAATAACTACAGCCTTTCGCAGCCTCGCCACTTTTGCAAGACGTGCCGCCGTTACTGGACTCGAGGGGGCGCTATGAGAAACGTTCCGGTGGGTGGAGGCTGCCGGAGGAACAAGAGGAGTAAGAAGTCCGGGGGAGCCGCCGCGGCTGTCTCCGAGAAACAAAGCGGCGGCGGCGCCGGGAATAGTAATAGTATTAATACTTCCACCGCCGCCCATATTTCATGCCCTACTAGCTGCGGAAGCGCAGAAATTTCCGGCAACTTTTCACAGCCGCCACTCATGGCCGCGTTTCAAGCCGGCTTAAACCATTTCGGCGGCTTCCAGCCTCCGCAACTGGTGGCTAACGAAAACGGAGGGTTTGGTGATATGAGCTTTCAATTAGGAGTCGAGCCTTGGAGATTGCCGGCGTCTTTAACCGCCTTTGACACGCCGCCACCCACAAATCTTTTCCCTTTCCAAAACGAAGCAATTGAGGCCTCATCTTCCCGCGCAACCCCCGAGTTTCCTCAGCTCACATCATCGGTAAAAATGGAAGACACCCAAGGGCTGAACAGTAATTCCACAAAGCAGTTCCTCGGCGCTCTTGGAAACAACCAATTCTGGGGTGGCGCCGGTGGCAACACTTGGACTACTGGAGGCTTTTCCGGCCACCTCAACTCCTCTTCCACTTCATCTTCCACCACCCATCTTCTATGA

>IbDof9

ATGGCATCATCATCACAGACACTGGAAAGCATGTTGGGTTGCACAAAAGCAGAGCAGCAAGAGAAGAAGGCGAAGCCATCTGCAGACGAACAATCATTGAAATGCCCAAGGTGCGATTCCACCAACACGAAGTTCTGCTACTACAACAACTACAGCCTATCTCAGCCCCGCTACTTCTGCAAGTCCTGCCGCCGCTACTGGACCAAAGGCGGCACCCTCCGCAACGTCCCCGTCGGCGGCGGCTGCCGGAAAAACAAGCGATCCTCTTCCCGCCCTCGAACTACCCAAGACCCATTATTCTCTTCACTCACCTCTACCCCCTTAATGCCCTCCCTCACCACCACCCTGCCTTATGACAGACCAATCATCTGCAATAATAATCCGACCAACAACGTTAATCCCTGCGATGTTCTTGAAAGCTATGGATTATTGGGCCATTTGGGCGCTGGATTTCTGCAAACTCCGGCGACAAACGGGCTGTTCCATAATCTGTGCTATGGGATTGGGATTGGGATTGGGAGTGAGCAGAGTAATAGTGTGGAATGGAATGGTGGGAATGGGGAAATGATGGGGAATTCATATCAAGATCGGATTTGTAGCGGTGGGGTGAAGCAAGAGGTGTGTTATGGAAGAGATGAAGGTGAGAGCAGGCTAAACTGGGGATTTCCATGGCTGCAGGGTGGGGAAGAAGGTGCCAACATGGTGGGCAACAGTGATGTAATTAGCTCAAGCAGACAAAGCTGGAATGAGAATGGGTTTGGCCCTTATTCTTCTTGGCATGGGCTAATCAACACCCCACTCATGTAG

>IbDof10

ATGGGTGAGCATCGGTCGGTTAACCGAAATTTTTGTACCCTTCCTATAACCGACCGAAATAACCGATCCAATGCTAGAGTTTTCGAGCAGCCGAGCAGGACAGCAGGTCAGGCGTCGGCGGTCGCGGTCGCGGTCGCGTCGAACCTCAACGTCAATTCCGTCGAAGCCTCGCAGAGTCGCAGACGGCAGAATAGTATACTCGTAGGTGAAGCCAGGCTCACGCCACCGCCGCTTCCAGTGTCAGTGAGCCGCACGTCATCACTCCACCGTAACTCATCTGGGGAGGAAGTCGCCGTTCGACAGGAGAAGAAGGCATCCCTTAGTTCACAACCTCCCTTCTGGCTGAGTGGTCTGATACTTCTCTCTCCTGGTGGCCCAGTTCAATTCCTTAGGGCTATAGTCCAATACGACGCCGTTTCACCGGCTGAGGATAACGGTGCCCGACTCGTCTTCATCATATGCGATTTCACCATTCCGTCTGCGAGATTCGACCATTTTCACCATTGTTCAACTAGATTCCCAGCTGACGTCGGCGGATCGAGCCAGGATTTGAATATTGACGTCACGAAACCCAATGTTTCACCGTCCAGGACTACTGACAACAAATCTCCCGACTTCGCAGTGGAGCCTCCTCCGTTCCGCTCGCCAAGTTCTCCATTCCTCTTGCCAAAAGCAAAAGGGAAGAAAGGTGGACCGGGGTGGCGGGGTGAGTTAATGGAGCAAGAAGCCTTAGGGGCTAGTGGCATGCAACCGAGGATGTGGATGACCGGACCATGCAGGGTAGACGCCAGGCTACCGGATGAAGGCAACGGAGTTGGTGATTTTCACCCGCCACTACCGTCGGCTAGGAGGTTGAGGATGATGGAGTACCCTGGCTCTCATCCTCCATCGAGACAATGTCCTCGTTGTCATTCTGATGACACCAAATTTTGCTATTTTAACAACTACAACATGAATCAACCAAGGTATTACTGTCGGGCATGCAAGCGCCACTCGACTCATGGTGGCATTCAACGTGACATCCCCATTGGCGGCAAGTCCAACAAGGGAAGGAAATCCACCAACAGGTACGAGAATAAGAGGGTCCAACCATCACTACCTCAACTCCAACCACCTTGTCCACAAGCAAATGTGGCTCCTCTAGCTTTCGGTCCTTTGGCTCTTCCTCCGATGGTGACACCTTATCGCGTTGAAAATGGGTATCTCAATATGGTAAACCCACTGAGAACTATTGAGCCACCATATAACTCAAGCCAAAACGCTTTCCAGCCTACTTCGCATTATGATTCAAGGTCACATAATTTATTTTTGAACAATAATGATGGAGCTAGTTCATCTAACCCCATTCCATTGAATGCCTCAACAGCAGCACCACTAGTGGATACACAAATATTGGTTGGGGTTCATTGA

>IbDof11

ATGACGATCTCTCTGACGCCGGCGCCGGCGCCGCCGCAAGACGACGGCGACGTTTCCAATTCTGTTACGACACCTTCCCATGACAACAAGAACACTTCAGGAGTAGAACTTATGGGCGATAAACAGGAAGTTGAATCCCCGGTTCCAATTGCTGAAGAGTCGGTAGAAGCAGTAACCTCTTCGGTTATTAGTGAAGATCCTCCGAGGACACAGAGTCCAGATAAAGGAACAGGGTCCTCGAAAGATATCAAGAAGGATGACCCGAGTGAATCGAGCGACTCTCAAGAAAAGACGCTGCTTAAAAAGCCAGATAAAATCCTTCCATGTCCGCGTTGCAATAGCCTGGACACCAAGTTCTGCTACTACAACAACTACAATGTCAACCAGCCCCGCCATTTTTGCAAGAATTGTCAGCGGTATTGGACAGCCGGGGGAATAATGAGAAACGTTCCTGTTGGTTCAGGCCGACGCAAGAACAAGAGTTCTGCTACGAATTACCATCATATAATGGTTTCTGAAAATGCAAATGGAGCTGTCCTTGCTTTTGGCTCGGATACGCCCCTTTGCAAAAATATAACGACTTCCGTGCTGGACCTTGTTGAGAAACCGCAGAATTGTGCTCCAAATGGAAGATATCGTGGATCTGAACAAGCTCCCGGTTGTTATGGTGGGAGGGATACTGGAAATGAACACTCTAATGGGCCTTCGAGTGCATCTATAGAGAGGGGAGGTAACGGGGCTGGTGTAACCGAATCAATGTGGAAGAACTTTCATGGATTTCCACCTCAAGTACCGTGCTTTCCCGGGCCTCCACCTTGGCCTTGTTCGTGGAATCCCGCTCTTAATCCTTCGAGCTTTCCCGTGTCGTTTTACCCAGCTCCAGCAGCGCCCTACTGGAGTTGTAGTCCTTGGAATGTGGCGTGGATCTCCCCGTCATCTTCTTCTGATCTCTCAGCCCAATGCAGTAGCCCGATGTCTCAAACATTGGGGAAACGTTCCCGAGAGGGACACATTCTCAAATCATCAAACAGCAGCACGGGAGAAGAGCCTTTAGGGAAGACGAGAGAGAATAGAGGGACGACGACTGTACTGATCCCTAAGGCTCTTCGAATTGACGACTCGAACGAAGCAGCAAAGAGCTCTATCTGGTCCACATTAGGAATCAAGAACGAAAAAACCGATTCACCAGGAGCAAGCCTCTTGAAGGCCTTTAGCTCAAAAGCCGACGAGAAAAACCCTCACGTGGCTGATCATTCTTGGTTGATGAAAGCCAACCCTGCCGCCTTGTCTCGATCACTCAACTTCCACGAGAGCACTTAA

>IbDof12

ATGGCGGAGCGAGCCCGCCTAGCCAACATACCGGCGCAGGACGCCGCCCTAAACTGCCCTAGATGCGAATCAACAAACACAAAGTTTTGCTATTTCAACAACTACAGCCTCACCCAGCCGCGCCACTTTTGCAAGTCGTGCAGGCGCTACTGGACACGCGGCGGCGCTCTCAGGAACGTCCCCGTCGGCGGCGCCTGCCGCCGCAACAACAAAAGATCAAACACCAAACCACCAAGTAATAGTACTAATAATACTCCCACATCTTCCACTTCTAGTCCTGCCTCAACTACCTTATTAGGCTTCTTGCCTCCCAACTTCAATCTCCCCCCTCTTCTCGGCGCCCAACTTTCCGATACCCATTTTCATGGCGGCGAAGGGGTCGGTTCTTTACTTTCCGGTAGCGCCGCCGTTGATCCGTGGCGGCTTCAGCAAGCTTCGCAGCGGCAAATGTCTTTGTTGGGCGGTCTGAATCCTTCGTCGTATGGATTATATCCATTTCAAGATGGTTCGGGTACGATGTTGGGACAAATGGCTTCCAATTCAGTGATGAAAATGGAACAAGGTAATCAAGAATCGAGATTGTTTTCACCTTGGTCGCAGATTTTTATGATTCCAGCAAGTGATGAACAGTGGAATTGTGTTGCCGATGTTTCTGCTAACCTTAACTCTTCTGCCAACATGTGA

>IbDof13

ATGGAGTACCCTGGCTCTCCTCCTCCACCGAGACAATGTCCTTGTTGTCATTCCGGTGACACCAAATTTTGCTATTTTAGCAACTACAACAAGAATCAACCAAGGTATTACTGTCGGGCATGCAAGCACCATTGGACTCATGGTGGCATTCAACGTGATATCCCTATTGGCGGCAAGTCACACAAGGGAAGGAAATCTACCAACAGGTACGAGAATAAGAGGGTCCAACCATCGCTACCTCAACTCCAACCATCTTTTCCACAAGCAAATGTGGCTCCTCTAGTTTTCGGTCGCTTTGGCGCTTCCTCCGATGGTGACACCTTATCGCGTTGA

>IbDof14

ATGGAGGAAATAGTGGGAACAAGTAGTGGCTCAAACAATAACTCATCTTCATCAAGTAAACCATCTTCTTCTTCTTGTGGGGAGAGAAAAATTAGGCCCCAGAAAGAGCAGGCTGTGAACTGTCCCAGGTGCAACTCTACTAATACTAAGTTCTGTTACTACAACAATTACAGCCTCTCACAGCCCAGGTATTTCTGCAAGACTTGCCGGAGGTATTGGACGGAAGGGGGATCTTTGAGGAATATTCCGGTGGGGGGTGGTTCTAGGAAGAACAAAAGATCTTCTTCTGTTTCTTCTGTTTCTGTTTCTGTTTCTTCTGCAAATAATAATGGTGGTTTAGCAGTGAAGAAGTTGCCTGATCTGGTGCCACCTGTCCCCCCAATTCTCCATCATCATCATCATCTCCATCAACAACAACAAAACCCTAGCAAGATCCATGAGAATCATCATCATCATCAAGATCTCAACTTGGGGTTTTCCCAGCATCAAGATTTCAAGACTATTAGTGAACTGATCCAGGTGCCAAACTACGAGAGCAAAGATGGGGGGAATGATCCTCACCGTCCATCTTCATCTTCATCACCACCATCCACTGCCACTCATCATCATCACCTCTCAGCTCTGGAGCTCTTGACTGGAATGACTACTGCTGCCACGTCATCATCAAGAGGACTCATGGGTTCTTTCATTCCCATCGCCCCCATTCCTGACCCTAATAATTACTCCTCTTTCCCTCTCCCCGAATTCAAGCCTTCCCTGAACTTCTCCCTGGATGGGATTGGGAATAATAATGGCGCCTATGGGAATCACCACCACCATCTTCAAGGCATGCAAGAGACAACCACCGCTAGCGGCGGCGGCGGAAGGCTTTTCTTTCCTTTTGAAGATTTGAAAAGCACCGCCGCACCAGACGGCGGCGAGCAAGAGAGAGACAATAACAACCCGGCTGGGGAATCAACCGGCGGCCATGGATTTTGGAATGGAGTCTTGGGAGCTGGAGGGGGTTCATGGTAA

>IbDof15

ATGGAGGAAATAGTGGGAACAAGTAGTGGCTCAAACAATAACTCATCTTCGTCAAGTAAACCATCTTCTTCTTCTTGTGGGGAGAGAAAAATTAGGCCCCAGAAAGAGCAGGCTGTGAACTGTCCCAGGTGCAACTCTACTAATACTAAGTTCTGTTACTACAACAATTACAGCCTCTCACAGCCCAGGTATTTCTGCAAGACTTGCCGGAGGTATTGGACGGAAGGGGGATCTTTGAGGAATATTCCGGTGGGGGGTGGTTCTAGGAAGAACAAAAGATCTTCTTCTGTTTCTTCTGTTTCTGTTTCTGTTTCTTCTGCTGCAAATAATAATGGTGGTTTAGCAGTGAAGAAGTTGCCTGATCTGGTGCCACCTGTCCCCCCAATTCTCCATCATCATCATCATCATCATCAACAACAACAACAAAACCCTAGCAAGATCCATGAGAATCATCATCATCATCAAGATCTCAACTTGGGGTTTTCCCAGCATCAAGATTTCAAGACTATTAGTGAACTGATCCAGGTGCCAAACTACGAGAGCAAAGATGGGGGGAATGATCCTCACCGTCCATCTTCATCTTCATCACCACCATCCACTGCCACTCATCATCACCTCTCAGCTCTGGAGCTCTTGACTGGAATGACTACTGCTGCCACGTCATCAAGAGGGCTTATGAGTTCTTTCATTCCCATGCCCCCCATTCCTGACCCTAATAATTACTCATCTTTTCCTCTCCCGGAATTCAAGCCTTCCCTAAATTTCTCCCTGGATGGGATTGGGAATAATAATGGCGCCTATGGGAATCACCACCATCTTCAAGGCATGCAAGAGACGGCCACCGCTAGCGGCGGCGGCGGAAGGCTTTTCTTTCCTTTTGAAGATTTGAAAAGCACCGCCGCACCAGACGGCGGCGAGCAAGAGAGAGACAATAACAACCCGGCTGGGGAATCAACCGGCGGCCATGGATTTTGGAATGGAGTCTTGGGAGCTGGAGGGGGTTCATGGTAA

>IbDof16

ATGGTTTTCTCTTCTGTTCCGGTCTATCTAGATCCTCCCAATTGGCCTAACCAGAGTTCGGCGCTACTTTCGGCGGCTTTGGTGGCGCAGGGGCAGCCGCAGCCGCCTGCGGCGGCTGGCGGCGGGATAAGGCCGGGATCGATGACGGAGAGGGCGAGGATGGCGAAGATCCCTATCCCGGAGGCGGCGCTGAAGTGTCCGCGTTGCGAGTCGACTAACACCAAGTTTTGTTACTTCAACAATTACAGCTTGTCGCAGCCGCGCCATTTTTGCAAGACTTGCCGGAGATACTGGACCCGAGGCGGCGCGCTGAGGAACGTCCCAGTCGGCGGCGGCTGCCGCCGGAACAAGAGGAGCAAAGGGAGCCGATCCAAGTCCCCCGCCCGAGCCGCCGCCGACGCGCCGAGCTCCAGCTCCAACAACGCTCATCGCCACGCCGATATTTTATCGGCGGCTCCGAGCCACCATTTGCCGCCGCCGCCGCCGCAGATACCGTTCCTCCCACCTCTCACCGGCTTCGCGCCGCCGGACCTCGGCTTAAATTTCGGCGCGAACGAAATGGAATTCCTGGCGGGAAACAACTCCGGCGGCGGCGGCATAAATTTATCCGCTCGGTTCGCCGATTTGTTCAGACTCCAACAAGGAATCACGCAACTCCCTTTCTTATCCGGCCTGGATCATCCCGCCGGCGGCATGTTCCAATTCGAAACCGGAATCGATCAATACGTCGCCGCCGGAGCCACAGATCATCTCGGAGCAAAACCCTTCGAAACCGGCGGCAATCTGGTAAATGTGAAGCTAGAAGAACACAATAACAACAATGCCTTAAATTTATCAAGAAACTTTCTGGGAATGTCAGGAAATGATCAATTCTGGAGTGGCAATAATGGCTTCGCAGCAGATCTGTCTGGTTATGCTTCTTCTACTGGCCGACTCTTGTGA

>IbDof17

ATGGTTTTTTCCTCTATTCCAGCTTATCTTGATCCAGCCAACTGGCAGCAGCTAAATCATCAAGTTGGAACAACGACACAGGTCCCTTCCGTGCCGCCTGCGGCGGCGGCTGCGCCGCCGCCGCCGCATGGAAGCGCGGGGACGATTCGGCCGGGATCTATGGCGGATAGGGCGAGGCTAGCGAACGTACCGATGCCGGAGACTGCTCTGAAATGTCCTCGGTGCGAATCGACGAACACGAAGTTTTGCTATTTCAACAACTACAGCCTCACGCAGCCGCGGCACTTCTGCAAGACGTGCCGGCGGTACTGGACGCGCGGCGGCGCGTTGAGGAACGTGCCTGTCGGGGGCGGTTGCCGGCGGAATAAGAGGAGTGGTAAGGGGAGTAGTAGTGGTGGTGGTGGGAGCGGTAACAATAATAATAAGTCTCCTTCTAATAGTAGTGAGCGGCAGGCGAGTAGCAGTTGCAGTGGCGTTTCCACTAGCAGTATTATGTCCGGAAATTCCGGGGGCGGGTTGTTGGGCGGGCCGCAAATTCCGCCGCTGCGGTTTATGTCGCCGTTAGGTCAACTCTCCGAGCACTACCCTACCGGAAATGATAATATTGGGCTCAATATCAATTACTCCGGTCTTTCCGCCATGGCAGGAGGAGGAGGGATTGCTTCGCTTCTATCAGGCGGTATTGAGCAGTGGAGGCTTCAAACACCGATCTTAGGCGGACTGGATCTGTCCCAACCGGGACTGTACCAATTCCCGGGAGGTTCGGAGGCGGCGCCGTCAGATTTTCTCGGCGGCGAGACGAGTGATACCAGGCCGAAGTTCACGAGTTCTTCGATGCTGACTACTCAGATGGCGTCGGTCAAAATGGAAGACCATAATAACAATCAAGGATCAAGTATGGCAAGACAGCTGTTGGGGTTTCAACCGGGCAATGACCAGTGGGGCGGCGGCGGCGCCGCCGCCTGGAGCGATGTTTCCGCTAGTTTCAGTTCTTCTTCCACCAGCAATCGTCTATGA

>IbDof18

ATGGATACTGCTCAGTGGCCACAGGGTATTGGGGTTGTGGAGAATGTTGTGGAAACAGCAAGAGTAGCAGAGAAGAAACCCAGGCCAGAAAAGGAAAAAGCATTGAACTGTCCTCGGTGCAATTCCACAAACACCAAATTCTGTTACTACAACAATTACAGTCTTTCTCAGCCCAGGTACTTTTGTAAGACTTGTCGAAGGTACTGGACTGAAGGGGGTTCCTTGAGGAATGTTCCCGTGGGGGGCGGTTCACGGAAGAACCGAAGATCTTCTTCTTCTTCCTTATCTTCTTCCTTATCATCTTCTTCCTCTACTTCTTCTAAGAAGCTTCCAGATCATTCCGCTCCATCGCCGCCGTGCTTTCCTCTAAACCCTAATGCCGGGCAGGATCTCAATTTGGCATACCCACCACCCAATACTTCTAATCACGGAATCTTGCACCATCTCAACCCGTTTCATATCCCCATACCCGTTTCCGACCCGTCCACCACCGCCGTGTTCTCTTCTGGATTCATGTTCCCATTGCATGATGTGAAACCCGCCGCCCTCGACGGGTTTGGGAACGGGTTTGTGAATCACGTGGAGGATAGTACTGGGGCTCGAGTCTTTTTCCCCAATCTCGAGGATTTGAAATCCGGGTTTGAGCAGTATAGAGCCCAAGGAGAAACGACTAACGGGTATTGGAATGGGGCCCACGGCGTAGCGGCGCCGCCGGCGGTGGCGGCGGCGGATGGTGAAACGGAGCAGTGTATGCATGTTTAG

>IbDof19

ATGACACCCAACAGAGCTCCGGCAGCGGGGCCGCAAGCCCGCCGGCGCCTCCGCGGCGGCGGCGGAGCAGGCGTTGAAGTGCCCCAGATGAGGTACTGGACGAGAGGTGGGGCTTTGCGGAGCGTACCCATCGGCGGCGGCTGCCGGAAAAACAAGAAAATGAAGTCTTCCTCTTCTTCCTCGTCGGCGTCCAGGCTTTCCGGTGACTCCACGGGCTCCTCCGGGATCGGCGGCGGGTTTAAATTCTTTAACAATCTCTCCCCCGCCATGGATTTTCAGCTCGGAGGGTTAAATTTCTCTACCCTAAACAACGTTAACTCTCCCACCGCCGCCATTTTCGAACACTACTCCACTTCCTCCCCGGTTATCCCATCAAACCCTTGTTTCAATCTTGACCCGCCGCCGGGAACCGCCGCAAACTCCGTTCTGGGTTTCAATTTTTCTTTCCCTTCTGTCCTAAAACGCCAGGGAGGAGAAAACACCTCGGCCGGGTTTCAAGAAATGGGCTCAAATATTCATCATCATCCTCAAGATAACAACAACAACAATAACAACAACCACCTGGCTTCTTCAATCGAGTCATTGAGTTCAATCAACCAAGAACTCCACTGGAAACTTCAGCAACAAAGGCTAGCCATGTTCTTTGGAGGAGAACACCAGAAAGAGATGATAAGCACTGCAAATAACTCATCCATTCCTGTTAACGAGGCCCAGATGATTCAGAAACCCCAGCCTATCTTGTTCCAGAATCTTGACATTTCTAAACCCGAAGCTTTTGATGGCAAAGACTGTGGAGAAAATGGGAGCGCGAGCTTAGCTACTGAATGGCTATTTGACAACACTTATGCCCAGGTAAATCCCAGTGCAACAAACAACACCACACCTGGAAATGGGAATGAAAACTCCACAAATTGGAATGGGTTTCAGGCTTGGTCCTCATCCAACATGAATCACTATAATTCCTTGCCATAA

>IbDof20

ATGCCATCAGCTGATACCAGTGATCAACGGCGCGCTGCCAAGCAGCAGCAAGTCGGCGCGCCGCCGCCGGAGCCGGAGCACCTCCGCTGCCCGCGCTGCGACTCAACGAACACTAAGTTTTGCTACTACAATAACTATAACTTCTCCCAGCCGCGCCACTTTTGCAAGTCGTGCCGCCGCTACTGGACCCACGGCGGCACGCTCCGCGACATCCCAGTCGGCGGCGGGAGCCGCAAGAACGCCAAACGCTCGCGCACCATCCCCAGCTCCAGCTCCGGCGCCGCCGCAGCCACAAACGCCGCCGCCTTCCCCGCCCACGACTTCCGCCACGCCGCCGCGCCATCCTCGTTCCTCTTCCCGCTCGGCGCCGATCACGCCGGCTCCGTGCCCTTCCCCGCCGACATGGTGAAGCCGGGCTTGAACGTGTGCGGGAGCTTCACTTCGTTGCTGAACACCCAGGGGCCTGGGCTTTTAGCGCTCGGCGGATTTGGGCTCGGGCCCACTATCGACGACATGGGCTACGGCTTCAGCCGAGCCGTGTGGCCCTTCCCAGGCGTCCCCGAAGCCGCCGCAAACGGCGGCACCGCCGCCGTCTTGAGCGGCGGCCCTTGGCAGCTCTCAACCGGAGAAAACGCCTTTGTTAACGGCGATTGTTTTGGCTTTGCCAGATCTTGCAATTTCTACCCAGGGAACCGTCGCCCTGTCATCGCCGGAAACATTAACGGCCGCGCTTGCTCTAAGGTTAACCGAAGAATGAGTGGCCGGGAAGTACACTTGAGGCTCTGTAGTCTAATCGACGATTCTCTCCGTCCGTATGTTGAGGTTATGCCTACTTCGTTTAATAAGGAAAATGTAAAGGAGCTATTAATCGCGCTCTCTCAAGTTTGCTCACAAATCAAGCGGTGGACTGTGGAATTCGCATCCGATTCTGATTCTGACGGCGTGGAGTCTGATTCCGATGCTGAAGTGACTGGAAATTCTGCTGGCGTTGGCCTTGTTCCTGGTTCAGAAACTGAAAGTCACTATAGTTTAGCAAAGGCCATCAGCGTCTTGATGGGCCTGCTTGCTATTGAATATCCATATGTGCAACACTTAGTGGGCAACATTCTGGTGGCTATTTCTGATTTCATAGTTGCATCTGGAAGTAGTTCGGATGAATTTATGCAGTTACTATCTCTCTTGAAGTTGGCAGCTGCTAGTTCTTTTCCATCTTCTACTGGATGTATAATGGCGGAGGCCAAGAATTCATGCTGCAATTCAGCAGCTTCTGTTTCTCCATTGAGTCCCCACCATAAAAGTGCAAAGTGGCTAACAGTGGCTGTCATTATTCAAGTTTTACGTAGGATACTAAAGAACTTGAAACAAGATGCCGATGATCACTTTTTCAAACTGTACTTGGGTGTGACGAACTCTTTTATTTCAAATATGCCATGGGATTTGATGGATCAGGTTTTTGTTAGTCAAACAAGTAAGGCATTGGGAGACCCCATTGCCGATGGTTTGCTTCAATTCCAACCTGCAAAATCAAAGTCAGCAATTATGTTTCTTGGAAATTTCATCCAGTTGTTGTGTTCCTTGGTTGAGAAAGATATTCCGATTGAAGCCGTTGCTGATGATTTAGATAAGCACCCACTTATTTGTGAGATCAGGAACATCCTTCCTAGACTCCTGGGTTGGTGCCTTGGGAATTTGCAAAACTCTGATGTCTATATCTCTAAATACTACAAGCACAAGCTCCTGATACTCATGATCAGGCTTAGTTTCAAAATCCAGTTGGATTGTTCAGTTCTTCTTTCATGGCTGCATCTCATCCACCTTTACTTTCAAGATCTCTTGTGCCTACCAATAGCTGGATTGGAGTCTGACAAGGATAAATATTTGGAAGGCTCACCATTCTGGGAAGATATTTTTGATGCAGCAAAAGAAAACATCTCATCCAGGCATTTGCATAGGCTGGCGATTTTTCTTTTTCTAAGATGTTCCTTTGGCTTGGTCAATATGGAGAAAAGTGGTCAAAACTGTGCATCCACAGATCTCAATTCTCACTCTGACCCGAATTTAGATATAGAGTGTTGCACTCAAAGCAAAGGTTTGCAAGAGCTCCATGAGTGGCTCCAACGTCTCTTTAATGATGTTTTTCTGGACCATGAGAAATCTGCTGAATCTATCGTGAGCTTTCAGTTTTCCTTCATCCAGCTTTATATGCATGAGGATGATATTTTGTTTCAAATGCTCCTGCAACTGCTTTGTGTCCCACTATGCTCTGAGAAATGGTCTGTTAAAGAAGGAAATCCATCCGCATATGATAACGCATATTCTATTGTTTTGCACCTTTTTAACCCTGTACATTTATTTCACATTTTTCTTGCAGAGATACACTATGATCATCAAGTGCTTCTTGATTATCTCATCTCAAAAGATACAGGAGCTAGTTCTGCTGAATATCTCTTGAGGTGCTTGAGCATGGTATTTAAGTCGTGGAGCTTATTTACAAATTTTGCGTGGAGTGGAAAGAAAGAAATCCAATTATACCAAAAGAGAAGAAAAGTTTCATTAGATCATCTGAGTTTTAAGGTGGAGGTGTCCACACCAGTGACAGATGGGCGTTGCTCACCGGTTGAACGAGATCAGAAGAAAGGAAAAGCATCTGGTGTCATTGACTGCAGAACTGAGAGGCTACCATTTCAGCATGCTACTAATTGTTTATTGGAACTGAAAACATCTATCGAAAACCTTCATCACAAGAACCTTTTTCCATATAACCCTAAAGTGCTTCTACAGCGGTGTCTTGTCAACGTTTCAGGAGCTCTGCCAAAAGCAGTAGGCTCTGAATCCAAATACTTGGTTGAAGAAATGGCTAAGATGAATGTCTCTGCCTGA

>IbDof21

ATGGAGCTCTCGAACCCGACCCAGACCCGAGCCCTGACTCGCCCTGTCAGATCTCCCTGCGAGCGAGCGAGTTTGTGTTTTGCAACGTTGGAAGTTGGAAAGAAGGAAAAAAAGTGGGCGCGAATGTCGGATTCGAGTAAAGACCCGGCGATAAAGCTGTTTGGGAAGACAATCCAGCTCCCGGAAATTCCGGCCTCGACGGCGCCGGCGACGGAGGGCTCTTGCCCGGACGATGCTCCCGGAGATGACAGCTCAGCTCGGCATGGCTCCGGTTCGGGAAATGACCCGGCTGAGGACGGCGGGGGCGGAGAAGAAGATGAACACCTGCAAAAGGAACCTGAAAGCTTCTATCTAGGGTTTATAGTATCTCTAGCCTTTTCGACCTTTGATCCAATCAAAAAAGGAGGAAAGTTAGATGAGAGTAAGGATGATGAATCTTTGATGGCAGAAGAGTTAACTGATCAGACTAGTGAGATGGAGACTGATGAGTTGAAAACGCCGTCTACCAATAAAGATTGTGGAGCTATGAAAAAGTCGGAGGTGGAGGAACAGGGGGAAACGGGTAATTCACAAGAGAAGGCTCTGAAAAAACCGGACAAGATACTTCCATGTCCGCGCTGTAATAGCATGGAGACTAAGTTCTGTTATTTCAACAATTACAATGTTAACCAGCCCCGCCACTTCTGCAAGAACTGCCAGAGGTATTGGACGGCTGGTGGGACGATGAGGAATGTACCGGTTGGTGCTGGCCGAAGGAAAAATAAGAATTCCCGGTTCCCATTACCGCCATGTGACCATCTCGGAGCCCTTTCCGAGTGCCCGAGTAGACCTTCCGAATGGAATCCAGCTTCCTACGATCAATCCCAATGGCACCCTTCTTACAACGGTTCGATAAACGGGTTCCACAAACTCGAGGAGCTTGGAGTTCCGGTCCCTCATGGAGCTGGAGATAATGGAGATGATTGTTCGAGTGGATCCTCAGTCACTGCTGCAAGTACCAAGGACGAAAGTGTAAAAAATGAAGTGCCAGGGAGACAGAACTGCCCGAACTTCGCTCCTCAGTTGCCATGCTTCCCCGGTGCTCCTTGGCCTTATCCATTGACTGCAGTTCAGTGGAGCTCCGCAATCCCTCCACCCGGTTACTGCCCTCCCGGCTTTCCTATGCCACCGCCTCTCGCCCCTCAACACCACATGCCATCAACTTCCGGTCCCAATTCTCCAACTTTGGGCAAGCATTCGAGGGATGAAAACATGCTGAAACCAGTCAACAGCGAGGGAGAAGATCCCAAAAAGGAAAGCAATCCCGATAAGTGCCTGTGGGTTCCTAAAACTCTCCGAATTGATGACCCTGAAGATGCTGCAAAAAGTTCTATCTGGGCAACGTTGGGAATAAAAAACGACAAAGCTGATTCAGTCGGCAGTGGCCTTTTCAAGGCCTTTCAGTCAAAAGACGACGAGAAGACTGATGCTTCAGAAAACTCGACTGTATTACAAGCCAATCCAGCCGCACTATCCAGGTCACTAAACTTCCACGAGAACTCATAA

>IbDof22

ATGGGAGAAGGGAAAGACCCAGGAATCACCCTGTTCGGAAAAAAGATTCCCCTACCGTTAAGGGTAGTGGTCTTCGCCGGCGACGAATCCGCCGGCGGTGATTCCGGCGGGGTTAATTCCCCTGATGAAAGCAATAGTGGGTCTGAGCGTGTTAGGTGTTTCGACGACGAGAACATAGAAGAAAGCAAGCGTGAAGATGAAATGGATCAGTTACCAGGAGAGGCCTCTGAAACTATCTCAGAAGAGAAAGATGATGATGAAGATGAAGATCAAAACATGGATGGCAAAGAAAGTTGTAAAGCTTTACAAGAATCAGGGAGCAATACTGAAACTCCCTCCACTGATGGCAACTCTCCAACAGCAAAATCGACCAAGACTGAAGATGATCAAACGGAAACAGACAGTTCTAAGGAGAAAACGCTAAAGAAGCCGGACAAAATTCTCCCATGTCCTCGGTGCAACAGCATGGATACAAAATTTTGTTACTACAACAACTACAATGTCAATCAGCCCCGCCATTTCTGCAGGAGCTGCCAGAGATACTGGACTGCCGGGGGTTCCATGAGGAATTTGCCAGTTGGGGCTGGTCGTCGCAAAAACAAGAGCTCTGCCTCTCATTGCCACATCACCATATCGAATGCTAATGTCTCTGATGCCCTCCACGCAATGCAGGCTGAACCTCCAAATGGATTTCATTACCCAGCTCATTATAAACCCAACGGCACTGTCCTGTCCTTTAGTCCTAACTCCTCTCTCCGTGAGTGTATGGGCTCCGTTTTAAATCCTGGTGAGACAAAGTCGCCAAATGGGACTCAAAATGGTTTCTCTAAACCCGACACAAGAAACTCTTCTTACAAAACCGGGAATGATTGCTCTAGCAAGTCTTCTGTCATAAATTCGAACTCAAAAGCAGAAGGGAGTAAAAACGCACCCCAAGACACAACGGTTATGCAGAATGTACATGCCTTCCCTTCTCCAGTTCCATACCTTCCTAAAGTCCCGTGGAATGCTGCAGCACCCCTGCCACCCATTTTCCCCTCGGGAGCTCCTGTCACATTCTGCCCTGCAACTTATTGGAATTATTGCCTTCCCGGTCCATGGAGTCTTCCTTGGTTAACTCCACCATCTCCAACAGTAAGCCAAAAGTCATCAATTTCTAGTCCTAATTCGTCATTGGGGAAGCACCCAAGGGACGGGGATTTACTTGAGCCAAACAATCCCAAGGGTAAAGAATCATCCGAACAAAAGAGCCCCGAGCGGAGAATCTTGGTTCCAAAAACATTGCGGATCGATGATCCCGATGAAGCTGCAAAGAGTTCTATATGGGCAACACTCGGGATCAAATACGATTCCATAAGCAAGGGAAGCATGTTCAATGCCTTGGAACAAAAAAAGCGATGA

>IbDof23

ATGGGTCTCACAAGTTTGCAAGTCTGCATGGATTCATCTGTTGACTGGTTGCAGGGCACAATCCATGATGAGGGTGGAATGGACTCATCATCTTCACCATCTGGTGTGGACATTCTGACATGTTCAAGGCCATTGATAGAAAGAAGGCTAAGGCCCCCCCATGACCAGGCACTGAAATGCCCAAGATGTGAGTCCACACACACAAAATTTTGCTACTACAACAACTACAGCCTCTCCCAGCCAAGGTACTTCTGCAAGACCTGCAGAAGGTACTGGACAAAAGGTGGAACCCTGAGGAACATCCCAGTGGGGGGTGGGTGTAGGAAGAACAAGAAAGCCTCTTCTTCTTCTAATAATTCCTCCTCTTCTTCATCATCTGCCAACAAGAAATCATCAAATGACCATAACCAATTCCCTCATCCCCCTCCTCCTCTTTATCATCATCACCAAAATCTTGGCCAATCATCATCCTCCACTTCTTCTCCTGGCTCTGCTGGCATTAACCCCACAGATCTCCACCTCTCATTCCCTGCAGAAATGCAATTCTCACACCATTTCACCAATTTCCACTTGGCCCCCCACACTGGTTTCTTGGGGATTGAGAGCCCCACCCCAATAGATTTCATGGAAGGGAAATATGATTCAGGGTTTGTGATGGGTGGTGCCACTTGTACTACTCCTCCTCCTCCTTCAAGAAACCAAGATTTTCTTGGGAATAACAATGGAGATTTTGGGATGATGGCTCAAACTACTTTCCCTACTTCTATTAGCCCTCAATTTGGAATGCCCATTGATGGGAACTCTGTGGGACCTACTCTAATGCTCCCCTATGATCATCATCATCATCATGGGGATCATCATCAATTCAATAATGCAATGCATCATCATAATCATCAAGATGTGAAGCCAAATCCCAAGCTTTTGTCCCTTGAGTGGCATGATCAAGGCTGCTCTGATGCTGGGAAGGATTCCTTTGGTTATCTCAATGGAGTTGGCTCTTGGACTGGCTTGATGAATGGCTATGGCTCTTCTGCAACAAATCCTTTAGTCTAA

>IbDof24

ATGGGGTTGAGTACTAAGCAGGTTTCGGGCGGGGATCATCATCAACATGGGATTATTGAGTGGGAAAATCCCCAGAATCCAGGTGGGAGTACGAGGTTAGATCTCCCGACCGTACAAGCTCAATCCGTACAGCCGCCGCCGCAAAAGGCGGAGCCTTTGAAGTGTCCGAGGTGTGCTTCGGAGAATACCAAGTTCTGTTACTACAACAACTATAATAAGTCCCAGCCGAGGCATTTCTGCAAAGGCTGCAAGCGCCACTGGACGGAAGGCGGCACTTTGCGTAACGTCCCCGTCGGCGGCTCCCGCAAGAACAAGCGCCTCAAGACCCCGCCGAACGCTAACGCCGTTACCCCCGCCGTTAAGTCATCATCTCCCGCCGCCGCCACGTGTGATAACCAAAACCCGGTTTTTTCCGGCGAGAACAAAAACCACGCGCCCAGTCTCTTCTTCCCCGCCGCCATCCAAGCCGACCCGGTAATCGGCGGCGCCGCCGCGGCGTTCAACATTGGCTCGGCGCCGATGGTTTTACACGACGATCAAGACACGACAAAACTCTTACCCTTTTCGTTCTCGAGCTTCTTCGATCCGATTTCTTGCTCGATTCCTTCTTTCAACACCCAATCCTCGTCCAACAACGCCTACAGCGACATCATAGTCGGAGAGCTCGATAATGTCGAGGAATCCACCATAACCACCGTCATGCCATTCACAAGCTCCGCCGTTATTTCTCAGCCATGGGACATGGCGAACTACTGGAGCTTAAGCGAGATTGATTCCCTGGTTTCAGCTGATCTCAATATTCCATGGGATGATTTAGAGATCAAGCCAAAGGGAAAGTGA

>IbDof25

ATGGTTGGAAGTAATTGTGATGAGAAGATGGCAGTCATCTCTTCCACTGCAAATGAATGGCCAATCCACCAGAAGAATCAGATAGATGGCAGAAGCTTGATGGGGTCTAGGGTTATGGAGAAACCAATGAACGGCCAAGATCAAGCGATGCCGCCGCCGCCGCCGCCGAACCAACAACCTCTGAAATGCCCACGTTGTGATTCATCGAACACCAAGTTTTGTTACTACAACAATTACAGTTTGTCTCAGCCCAGGCACTTCTGCAAGGCGTGTAAGCGGTACTGGACTAGAGGGGGGACCTTGAGGAATGTTCCGGTGGGGGGAGGGTGCCGGAAAAACAAGAGAATCAAGAGGCCATCTGCGCCGTCCGTTTCATCGTCATCAGCTACACAAGATCACCACATCTCAGCTCCTTCATCTTCAGCTCCAAATCTTCAAGTTCAAGCCCAGATGGATCATCATATTAATCCCTTGCTTTATGGGCTCCCCACTAGTACCCCTTCTGAGCTAGGCCTCTCGTTTCCAAGATTGTTCACCTCTAGGGTTTCAAACTTTGAGGGTACCAATATTCCTGGATATGATCATCATCACCATATGGGATTAGGGTTTTCTTCTTCTTCTAATTCTGGGCTTTTGGGTGGTGAAAATGGGTTTCCTAATAATAATACCTCAAATAACTCTCTTCTTTCTAGCTTCCATGGCGGATTGTCTTGTTCTTCTTCTTCTTCTAGCTCCAGTTCTACTTTGGCTTCTTTGATTGCTTCTAGCCTTCATCAAGGGGCAAACAATAATGGGTTTCATGGGTTGTCCCCATATGGAGAGATGCAAGTGGGAAATGGGAATATGGGGCCAAAAGAAGTGAAAATGGAAGGTAATTGGAATAATAATAATAATAATAATACTAATAGTTTGTCTAGCCACCACTATCAGATCATGAATCAGACAGCCCAAATCAACACTTCTTCTTCTGATAATCCTTCACTTCCATGGAATAATGGTGGTTCTTGGTTTGATCCTTCCAATATGGGATCTTCAGTCCCTTCAATTCTCTGA

>IbDof26

ATGGCAGAGGTTGAAGACAGCCACAGCGCACAGGGAATCAAGCTGTTTGGGGCGACAATAATAACTGTCCAAGAAAAGAAATCCGAGGACGAGAAATCCGAGGTCAAAGGTGACGATGATCATGGCAGCACAGCGGATAATCAGAAGAGGCCCGACAAGGTGATTCCATGTCCGAGGTGCAAGAGCATGGAAACCAAGTTTTGTTACTTCAACAACTACAATGTTAACCAGCCCAGGCACTTCTGTAAAGGCTGCCAGAGGTACTGGACGGCCGGCGGAGCTCTCCGGAACGTCCCCGTCGGGGCCGGCCGCCGGAAAAACAAGCCGCCGTGCCGAGGGATGATAGACGGGTTATCGGAGGGTTGCCTGTTTGATGCTTCTGGGTTGCTGCAAAATCTTGACTTTGATGGGGCGGTGGTGGAGGAATGGTACGCGGCGGAGCAAGGCGGCGCCGGAGTCGGAGGTTTTCGTAATCTTCTTCCGGCCAAGAGGAGACGGAAGATCTCGGCTGGTCAACCTTGTTGA

>IbDof27

ATGGCAGAGGTTGAAGACAGCCACAACGCACAGGGAATCAAGCTGTTTGGGGCGACAATAATAACTGTCCAAGAAAAGAAATCCGAGGACGAGAAATCCGAGGTCAAAGGTGACGATGATCATGGCAGCACAGCGGATAATCAGAAGAGGCCCGACAAGGTGATTCCTTGTCCGAGGTGCAAGAGCATGGAAACCAAGTTTTGTTACTTCAACAACTACAATGTTAACCAGCCCAGGCACTTCTGTAAAGGCTGCCAGAGGTACTGGACGGCCGGCGGAGCTCTCCGGAACGTCCCCGTCGGGGCCGGCCGCCGGAAAAACAAGCCGCCGTGCCGAGGGATGATAGACGGGTTGTCGGAGGGTTGCCTGTTTGATGCTTCTGGGTTGCTGCAAAATCTTGACTTTGATGGGGCGGTGGTGGAGGAATGGTACGCGGCGGAGCAAGGCGGCGCCGGCGTCGGAGGTTTTCGTAATCTTCTTCCGGCCAAGAGGAGAAGGAAGATCTCGGCTAGTCAACCTTGTTGA

>IbDof28

ATGAGACCAATGGAGGAAAATCAGGAAATAGTACCAATAATTGGAAACACAAGGGGTGAGAGAAAAGGTAGTATTAGGTTTCAGAAAGACCAGGTTTTGAACTGTCCCAGATGCAATTCTGCAAATACTAAGTTTTGCTATTACAATAACTATAGCCTTTCACAGCCAAGATACTTTTGCAAGACTTGTAGAAGGTATTGGACTGCAGGTGGATCTCTGAGGAATATTCCTGTGGGTGGGGGTTCAAGAAAGAACAAGAAACCACTCAAGAAAGTGGCTGATCTTGTGCCACCTGTCCCAATTTCTGGTGATCACCAAAGCTCTAGCAAGATCCATGGGGGTGAGGGTCATCATCAAGATCTCAACTTGGGAGGTCAGCTTTCAGCTATGGAGTTGTTGACTGGGTTCAGTACTTCAATAGGGTTGAGCACTGCTCCTTTCATTTCTGATCCAAATTTGCTGTACTCTCAAGCTGGGATTTCTTTGCCTGAATCCAAACAATGCCTAAAGATCTGTTTGGATGGGATTGGGAATCTTCAACCAATGCAAGATACTACTACTAATACAAGGTTTTTGCTTCCCTCTGATCAAGATTTGAGAAGCACTGCAAGTGATGATAATGGAGTCTTGAGAGGTGATGGAGGAGGAGAATCATGGTAA

>IbDof29

ATGTTGAGTAGTGAGAGTAGTCCAATGTTGGACTGCCATTCAATTAGGCCAATTCTTATGGATAGGAAGTGGAAACCCAACATTGAGTTGGCTCCAAATTGTCCTAGGTGTGCCTCTACCAACACCAAATTCTGTTACTACAACAACTACAGCTTGTCCCAGCCTAGGTACTTCTGCAAAGGTTGCCGCAGGTATTGGACCAAGGGCGGCTCCCTCCGCAACGTCCCTGTCGGCGGCGGATGCCGCAAAACCCGCCGCTCTAAATCCGCAAGGCAAGCCGCCGAGCAACACCGTTCCAATGCCCACCACGCGCCTGCAGGCGCGTTGGACAACGGGTCGGCCGGCGCTGAAGAAATTGATATGGCCGCCGTTTTTGCAAAGTACTTGAATCAAGGCGATCTTTCTCCCAGCGGCGCTACTAGCTCCACTTCTGCGTCAATGGCGCTCAGCTCGTTGGACTCGGAAAGCCAAGTGGATGAGCTGTTGTTGTTGGATTATCAAGAAGACCCTTCTCCCCTGTTCTTGGATCAAGCTGTTGAGCTACAGGAATCCAGCCCTCAAGCCTCCATCAACGTTCAAGAACATCTTCTTGATTACAACCAGAGCGCACTCGACTTGCAAGCTCTTCTTGAGGATGATCAGTGGCCCAATTTTGCATGGCAACAACCCATGATTCAACAGCAAGATCTCGGGACATTCTTCGGAGATAATGATCTTGTTTATCCGACCAAAGCTTCGACAACTCTACCTAACAACGATGTCTGGGGATCATTTGATCTGTCTGGTTGTGAAATTTTACCCAGACCTTGA

>IbDof30

ATGTTGAGTAGTGAGAGTAGTCCAATGTTGGACTGCCATTCAATTAGGCCAATTCTTATGGATAGGAAGTGGAAACCCAACATTGAGTTGGCTCCAAATTGTCCTAGGTGTGCCTCTACCAACACCAAATTCTGTTACTACAACAACTACAGCTTGTCCCAGCCTAGGTACTTCTGCAAAGGTTGCCGCAGGTATTGGACCAAAGGCGGCTCCCTCCGCAACGTCCCTGTCGGCGGCGGATGCCGCAAAACCCGCCGCTCTAAATCCGCAAGGCAAGCCGCCGAGCAACACCGTTCCAATGCCCACCACGCGCCTGCAGGCGCGTTGGACAACGGGTCGGCCGGCGCTGAAGAAATTGATATGGCCGCCGTTTTTGCCAAGTACTTGAATCAAGGCGATCTTTCTCCCAGCGGCGCTACTAGCTCCACTTCTGCGTCAATGGCGCTCAGCTCGTTGGACTCGGAAAGCCAAGTGGATGAACTGTTGTTGTTGGATTATCAAGAAGACCCTTCTCCCCTGTTCTTGGATCAAGCTGTTGAGCTACAGGAATCCAGCCCTCAAGCCTCCATCAACGTTCAAGAACATCTTCTTGATTACAACCAGAGCGCACTCGACTTGCAAGCTCTTCTTGAGGATGATCAGTGGCCCAATTTTGCATGGCAACAACCCATGATTCAACAGCAAGATCTCGGGACATTCTTCGGAGATAATGATCTTGTTTATCCGACCAAAGGTTCGACAACTCTACCTAACAACGATGTCTGGGGATCATTTGATCTGTCTGGTTGTGAAATTTTACCCAGACCTTGA

>IbDof31

ATGTCGACGGAAGTTAAAGATCCGGCGATCAAGCTGTTCGGAAAGACGATTTGTCTGCTTCGAGATGACACCCTCCCCTGTTCTATTGCGGCTGATCAACCTGCCAAGCGCAGCCGCTCCTCGATCACCACAACTTCGCGTGACTCCAAGAACACATCGGGAGAAGTTTCAGAGAGTCAACATCAGGACGATGAATGTAGAAATCCGAGTGGCGAAGATTCTGTCGGAGCAGAAACCTCGTCGCGTACGAGCGATGACGACAAGGCGGCACAGAATCCAGACAAAGAAACACAATGTGCAGCGGGAAAAGATGTGAAGAAGGACGATGAAAGCGAGACAAGTGATTCGCAGGATCAGAAGAACCTTAAAAAGCCCGATAAAATACTGCCGTGTCCGCGGTGTAACAGCAAGGACACCAAGTTCTGTTACTACAACAACTACAACGTCAACCAGCCCCGTCACTTCTGCAAGAACTGTCAGAGATACTGGACCGCCGGCGGATCCATGAGGAACGTGCCAGTGGGTTCCGGCCGCCGCAAGAACAAAGCTTCAGCCACCAATTTCCGCCACATAATGGTTTCGGACGCCCTCCACGCCGCTCAGTTCGCTTCCTTGAAACCAAATGGAACTGTCCTCACGTTTGGCTCCGACAGAGCAGTTAGTAATGAATCTATTATTCCAAACGCCTGGAATTCTGCTCCTCCTCCCCTGTACGGCGTGTTGGCTCCCTGGAGCGGGCGGTGGATCTCTGCACCGGCCTCCCCTGATCCATCAACTCCAACTTCCCCGAGGAGACTTAAAATGGAGGATCAGGCAATCTGGTCTACACCAGGAGGAGGAGGAGGAAGTCTGTTCAAGGCGTTTAACTCAAAGGATGGTGAGAGAGACAGAGATAGAAACCATTCTCTGAGTAAGGGGTTCACTCGGGTCAGCTTCAAAGCCGTACATGTGCGCCGAGTTTGGCGCCAAGAGAAGAAAGTATTAGTTATGAAGAAATTCAACATTATTCTAACGGTACCGGAATTTTCCCCCGATTCTGCCGGCGTACAAACCCCGCCGGTATCTCTTCTCAACCTCACATTCCGGTTCCCCGCCGCCCCGCTTCCGTTATTCAGATTCCATTTCCTTCTCCAAATTCCTCACAATTTTGTCGAATCGAATCGAACTTATGCTTTAGGAGCTTGCGAGGTAGAACTTTGCAGTGGACATCAGTTCTTTGTTCAACAGAGCTGGTGTCCTCAACATATATCAATCCCATTAAAAGTTCAACAAGCTTACGGCTTAATGGGGCGGAACCGGTTACCGTTAAAGAGACTGACTAGTGCCAACCGGAGGATAGCCTACTCAAGACGAAAGAAGGGTCTGATGAAGAAAGCTAGAGAATTGTCTGTCTTGTGTGATGTTGAAGTGTTTCTTGTCACATTTTCACCTGGTGGTAAACCTACTGTATTCATTACAGAGAATAGTTCCATTGAAGATGTGATTGAGAAGTTTGCACAACTGAAGCCAGAAGAAAGGGCAAAAGAGAAACTGGAGTGCCTTGAAGTGGTGAAGAAAGCTTGCAAAAAGTTTGATCACCATGTAGACATCGGGGAACTTTTTTATCCAGGGGATCTTTCAGATGAGGATTTGACATCTCTGGCTGATTCGTTGAGAACTCGATTTTCTGACACCCAAAACAGACTCAGCCATTGGATGAACATAGACAAGATCAGTAATACTGATCAACTTGGGAAAATGGAGGAATCATTAATCAACTCACTCCAAGACATTCAGAGACATAAGAATGGTCTTCTTGCTCAACAACTGAAATTGCAATGCAGTGATGAGTTGCAACATGTGACAGATTTTCCAATTGGTATGGGTACAAGTCAAGTGCTCCAGCCTTCTTCATGGACACAAAATGGTATTAATCAAAACATCAACTTCGACAAGGACCTTAATTTAACCCAAGGCTATCACAGATGTATTGTCTCCAGGCTTCCAGAGTGTTCATTTGAGAGCTATACAAATCTTTTTTGCTTTGATAAAGAAGTGGAGGTGACAAGACCCAGTGGAGAAGATAGGACTAATCCACTTCTGGACTATGAACACATGCAACATTATTTTCAGTTCCAATCGGCCGAAGAAAATTTGGTTAGTTGCTTCCCGTACCTGTCTCCAGGAACAACAGAATTCCCTAGTTTTGATGTTACAGACAAGAGTTTAGAAGAATTATTTGAAAATAGTGGGAATCATTTTTCCGATAGATTTCTTAGCAGCTATGGAGTGGATTCCCAGCTTCTGAGGCCACTGGATGCTAGTTTTCATGAATATACTGCAAAATACATCAAAATATTGCAGGGCTTAATGGGCCAACAGAAGTGTTGGGCCATTTTGCTCAATTGTCCAGATTTCAATCTAGTCTAG

>IbDof32

ATGATGGAGGGAGCGCCGGCGACATGGAAGCCTAACGTTGAGATTTCTCCGACCTGTCCTCGTTGCGGTTCAATGAACACCAAATTCTGTTACTACAACAATTACAGTTTGACCCAACCTAGGTACTTCTGCAAAGGTTGTCGGAGGTACTGGACCAAAGGGGGTTCTCTCCGCAACGTTCCCGTCGGCGGCGGCTGCCGGAAGACCCGGAGAGCCAAATCCTCGTCTATTCGCGTTGTCTCCAATCATCGCCGAGGAGGGGTGTTTGGGATCTCCGCCGCCGACAACAATAATCCCGGCGGATCCACTACTAGTCCCACCTCCGCCGCCGCCGGCGGGACTAATGGCCCCGCCCCCAACATTGATCTCGCCGCCGTTTATGCGAACTTCATGAATCCTAATCCTCAACCTCAACCCGCCCACATCCCCGAAACTACACTGCCTAATAATAACGACGGCGGCGGCGGCGGTGGCGCGGATCCATCGTTTGAATTTTCCGGCTATCCGGCCATGTTGAACGTGGATTATTTCGTCCCCGAGGCCGCCATGGCTCCTCAAGACGGCGGCTTCGTCGTCGACGAATTTGGGAACAATTTTAATAATTCCGGAGCAGCGATGTTTCAGGAACAATTCTGCGGCGAGTCGTTGCCCCCAATACTCCCTCCTCATGAAGAGTTAACAACCTCGGAAGGGTGGCCCACCCACAATTCCGACATGAATATGAATATGATGTTTCCATTGCAGCACACAATAAGTGCTCATCATCATGAACCAGAGCTACAAGGTTGTCCTAACCATCACACCACTTCAAGTTCAAGTCTTTTCAGCATCCCCACCACGTATGATTCCATTTTCCGGCCTTAA

>IbDof33

ATGATGGAGGGAGCGCCGGCGACATGGAAGCCTAACGTTGAGATTTCTCCGACCTGTCCTCGTTGCGGTTCCATGAACACCAAATTCTGTTACTACAATAATTACAGCTTGACCCAACCTAGGTACTTCTGCAAAGGTTGTCGGAGGTACTGGACCAAAGGGGGTTCTCTCCGCAACGTTCCCGTCGGCGGCGGCTGCCGGAAGACCCGGAGAGCCAAATCCTCGTCTATTCGCGTTGTCTCCAATCATCGCCGAGGAGGGGTGTTTGGGATCTCCGCCGCCGCCGACAACAATAATCCCGGCGGATCCACTACTAGTCCCACATCCGCCTCCGCCGCCGCCGGCGGCGGGACTAATGGCCCCGCCCCCAACATTGATCTCGCCGCCGTTTATGCGAACTTCATGAATCCTAATCCTAATCCTAATCCTCAACCTCAACCCGCCCACATCCTGGAAACTACACTGCCTAATAATAACGACGGCGGCGGCGGCGGCGGCGCGGATCCATCGTTTGAATTTTCCGGCTATCCAGCAATGTTGAACGTGGATTATTTCGTCCCGGAGACCGCCATGGCTCCTCAAGACGGCGGCTTCGTCGTCGACGAATTTGGAAACAATATTAATAATTCCAGAGCAGCGATGTTTCAGGAACAATTCTGCGGCGAGTCGTTGCCCCCAATACTCCCTCCTCATGAAGAGTTAACAACCTCGGAAGGGTGGCCCACCCACAATTCCGACATGAATATGAATATGAATATGATGTTTCCATTGCAGCACACAATAAGTGCTCATCATCATGAACCAGAGCTACAAGGTTGTCCTAACCATCACACCACTTCAAGTGCAAGTCTTTTCAGCATCCCCACCACTTATGATTCCATTTTCCGGCCTTAA

>IbDof34

ATGTTGGGGTGCACAAAAGCACAGCAAGAGAAGAAGCCAAGGCCAGCAGATCAGCAAGCACTAAAATGCCCAAGGTGTGAATCCACCAACACAAAGTTTTGCTATTACAACAACTACAGCCTGTCCCAGCCTAGGTACTTCTGCAAGTCATGCCGCAGGTATTGGACCAAAGGTGGGACCCTCCGCAACGTCCCCGTCGGCGGCGGCTGCCGGAAGAACAAGCGCTCCTCGTCCTCGGCTTCGTCGAGGAGCCGGAGCCAGGACCAGTCCTTGTGTTCCAGTCCGGTCCCGTTGCCATCCTTGGCTGGCTTGCCTTACGAGGCCAGCGATCTTAGCCTGGCATTGGCTAGGCTACAAAAGCAAGCCAACCTAGGGGTAGGGGAACATGAGATGGGTATGATGTGTAATCCCAATAATACCCCTTATGACATCGTCCTCGGAAACCACCACCACGGGTTTCTTGAAACGGCGGCGAACGCATCGTTCCATAATATGTACTACGGGAACATCAACGTGGGCGGCGAAATGGGGATTCCGTACGAGGAACACCACCTCGGCGGCGGAGGCGCGGCGGCGGCAGTGAAGCAAGAGATGTGCAGTGCGAGAGAAGAAGGGGAGAGCAGCAGGGTTTTATGGTCATTTCCATGGCAGCAAGTCGGAGCAGATCATCAAGGCAACAACAACATGGGCGATCAGATTGATTCAAGCAAGCAAAACTGGTATGGGTATGGGAATGGGTTTGGGTCATCTTCTTCTTGGCATGGACTTCTCAATAGCCCTCTCATGTAG

>IbDof35

ATGCAAGATGTTCACCCAATTAGCGGCGGTGGAGCTCGGATGTTCGCCGGTGGAGGTGATCGGAGGTTGCGGCCACACCACCACCAGACGGCGGAGCTGAAGTGCCCCCGCTGCGATTCGCTCAATACCAAGTTCTGCTATTACAATAACTATAACCTCTCCCAGCCGCGCCACTTCTGCAAGAGCTGCCGCCGCTACTGGACCAAAGGCGGCGTCCTCCGCAACGTTCCCGCCGGCGGCGGCAGCCGGAAAACCAAACGCTCCAAGCAGAAATCAGTCGATTGCCGCAGCGGCGGCCACCTGAGTCTAATTCCCATTCCAGTAGCGAGAGTTCTAGCATCACGCGCCGCCGCCGCCGCGGCGGCTGCGGCGGAGCTCGGGAACTTCACGACCCTGATGACGTCATCGGACGGCCCGTCGTCTCTCGTCGAGTTCACCAACGCTGCCGCCGCCGCCGCCGACACGTTCCGGTTGCCGCACAGCCCTAAAGCTCAGTGGGGCCCGCAGCCAAAGATGGACGGCGAGGATGGAAAGATGCAGGACATCACGGCAGCCGGGTTTTTAGACGATGCTACGCAGAGCAGGAGAAGCAACGGTGGACTCTCACCGTTGGATTGGCTAATCGGAGGTGGCCAGGGCCACGGGCTGTTTGATCTTACCGGAGCCGTTGATCAATCGTACTGGAATGATGATCACACCCTTAATTACCTCCCTTTATGA

>IbDof36

ATGTCAAAACTCCCCCCTGCTGATGAAGACTCTCCTTCGCCAAAAACTTCCAAGACTGAAAATGATCAAACTGAAACAAACAATTCACAGCAGAAAACTCTGAAGAAACCTGATAAGATTCTCCCTTGCCCTCGTTGCAATAGTATGGATACAAAGTTCTGTTACTACAACAATTACAATGTCAACCAACCTCGTCACTTCTGCAAGAGTTGCCAGAGGTACTGGACTGCCGGGGGTACCATGAGGAATGTGCCGGTGGGGGCTGGTCGTCGCAAAAACAAAAATTCTGCGTCTCATTGCCGTCATATTACCATCACTGAAGCCCTCCAAGCAGCACGGATTGATGTTCCAAATGGATTTCACCATCCAACATATAAACCCAATGGCACTGTCCTGTCCTTTGGTCCTGAATCACCACTTTGTGAGTCTATGGCATCTCTTTTGAATCTTGCTGATAAAAAGGTGCCAAATGGGATGCCGAACGGTTTCTACAAGCATGAACAGGGGAATTCTCCTAATAAAGTTGGAGAAAATGGGGATGATTGCTCTAGTGTGTCCTCAGTCACTACTACAAGTTCTATGGCAGGAGGTAAAAATCCTCCCCAAGAGGCAGTTATGCCAAATATAAATGGCTTTCCTACCCCGGTTCCATGCCTTCCTGGAGTCCCCTGGCCTTTCCCATGGAATGCGGCAGTTCCTCTGCCAGCCATTTGCCCCCCTGGATTTCCTATGCCGTTCTGCCCCGCACCTTATTGGAATTGCGCAGTTCCTGGTCCATGGAGTCTTCCTTGGTTGGCTCCACCATCTCCAACCGCAAACCAAAAAACATCAAGTTCCTCTCCTAATTCTCCCCTAGGGAAGCATTCTAGGGAAGGGGAGTTGCTTACTCCCAACAATCCCGAGGCTAAAGAATCATCTGAACAAAAGCTCTGGAAATTCAGTTTTGGGGTGGGCTTTTCAAGGCCTTGCAACCAAAAAGTGATGAAAAGAGTCACACAGCAACTCCTCCTTCAGCATTGCAGGCTAATCCTGCAGCACTATCTAGATCTCTCAGCTTCCAGGAAAGCGCCTAAAGTGGGACTTGGAAGACTGAAAGCGCCAACAGTGGGATATAAAACGTGCAGAGACGAGACGCTAAATTCCCTGAAAGCCACAGCCTCTAGTTAA

>IbDof37

ATGGTTTTCTCTTCAATTCCTGCTTATCTTGATCCCTCCAACTGGCAACAACAGCAACTGAATCATCATCTTCAAGGGGGGAGTAGTGGAATTCCAACTCCTCACCTGGCGGCGGCGGCGCCGCCGCCGCCGGTGGTTGGCGGTGGAGGAGGATCGATCCGGCCGGGTTCCATGGCGGAGCGGGCGCGGTTGGCTAATATGCCGACGCCGGAGGTGGCGCTAAGGTGCCCTAGATGTGAATCCACGAATACGAAGTTTTGCTATTTCAACAACTATAGTCTCTCGCAGCCGCGCCACTTTTGCAAGACGTGCCGCCGATACTGGACCAGAGGCGGCGCGTTGCGGAGCGTTCCGGTGGGCGGCGGCTGCCGGAGGAATAACAAGAGGAGCGGGAAAGGCGGCGGCGGCGGAGGCGCGAGTAGTAGTAGTTCGAAGTCTACTTCCTCCACCACCACCGCGAGTAACGATCGCCAGCAAAGCAATAATTCCGGCACGGTTTCCGCGGTGTCGGGGCCCACCACCGCCCATAACTTACTCGGCCTTTCACCGCAAATCCCGCAACTACCTTTCATGTCGTCGTCCCCTATCCCCCAACTCTCCGACCATCACTACGCCGCCGGAATTTCCCCTGCCGCAGATAATCTCATCGGCGGCGGCGGTGGCGGCGATAATCTCCTAGGCGGCGGAGGCGTCTCCCCAACTAGCTTCATTGGCGGCGCTCCATTTAGGCCAAGGATTTCTTCCGCCATGTTAGCTCAACTCGCAGCGGTGAAAATGGAAGACACTAACAACAACAATCACACCATCAACAATCAAGAATCGAGCTTGCCGAGACAGATTTTGGGGATTAATAACAATATTATTAATCCAGGTGGGAATGAGCATTGGAGTGGCGATAACAATGGCGGCGGGGGAGGTTGGAGTGACATTTCTGCTAGTTTCAGTTCTTCTTCCACAAGCAACACTCACTTGTAA

>IbDof38

ATGATGAACAGTTATCAGACAGATGGAAAGAGCTTGATGGCTTCTTCTTCTTCTTCTTCTCCCTCATCATCAACTGCTGCTAGGGTTATGGAGAAACCGATTAGCCAATCAGAACAACAACCTCTGAAATGCCCACGTTGTGATTCCTCGAACACAAAATTTTGTTATTACAACAATTACAGTCTGTCTCAGCCCAGACACTTCTGCAAAGCTTGTAAGAGGTATTGGACTAGAGGTGGGACTTTGAGGAATGTTCCAGTGGGTGGAGGGTGCAGGAAGAACAACAAGAGAATCAGGAGATCATCTTCTTCTTCATCGTCCCCAGCCGCCATTGATGTCGTCTCTTCTTCATCAACCTCTCCTGCAGTTTCTTCAGCTCCACTGCAAATTCCGGCTCCCCAGACCAAACCCCAGATTAATCCTTTGTTTTATGGGCTACCCATCAACCCTAATTCTGAGCTCAACCCCCCATTTCCAAGATTGTTCGGTTCTAGGGTTTCAAGCAGTGAGAGTTTTGATAATAGCAATCATGATAGTAGCATGATCTCTTCTTCACCTGGGCTTTTGGGTGGCCATGGAGAGAATAATGGGTTTAAATTTGTTAGCTCACTCATCATCAAATCCTCTTCTTTCAAGCTACCCACTTCGAGCCTCGAGCAACGAAAGTTCAATGCTTCTGGGGACATTACTAACAGAAATTTTCCGGGCATGCCATCTTATAATGGAGAATCAGTAATGGCAGGGAATATGATTCTTGAAAATGAGGAGTTCTTGAAAGGTGAGAGCCAAAACAGGTTGAATTGGAACGGTGGTGTTCCTATGAATCCTATGAACAATCACAATCTGCATACAGAAGGGGTGGTGAATTTTAACTATTCAACACCCTCATCAGATCCTTCATTTCCATGGAATGGTGGTTGGATGGAGCCTTCTCTCATCTAG

>IbDof39

ATGCAAGATATTCATTCGATTGGCGGTGGAGGAGGAGGGCGGTTGTTTGGCGGCGGCGGCGATAGGAGGCTCCGGCCGAACCAGCATCACAACCTGCCGGCGTTGAAGTGCCCCCGATGCGACTCGCTCAACACCAAATTTTGCTATTACAATAACTACAACCTCTCGCAGCCGCGTCACTTCTGTAAGAGCTGCCGGAGGTACTGGACCAAAGGCGGCGTCCTCCGCAACGTCCCCGTCGGCGGCGGCTGCCGGAAGAGCAAGCGATCTAAACCTAAATCGACCGCTTCTGCTTCGGCTTCGGCTTCTACGCCTGCCGACGCTTCGCAGGAGTGCAAATCGAATTCTCATTCCAGTAGCGAGAGCTCGAGCCTCACCGGCGCCGGAACCGCCTCCGCCGCCGCCGAAGTCGCGTCGACGAGATATTGGAACCTTCTCGAACATGATCACTCCCTCCAACGAACCCTCCCTATTAGGGTTCGCCAATATGGCCGACATTTCCCCGACGTTCCAGATTCAACAGAGCCAGCCAGGGCAGGACGCTCACTGGCCGCAACCGGAAAAATAGCCGATCAGAATTTCAATCCGCCGGAGATCACGGCGGCAGGGTTTTTAGATCAGACGTCTCAGATTGATTTCCCCAGCTTGAACCAGCAACAGAGCAGGGAATCCAACGCCGGCCTTCCTCCACTGGATTGGCAACCCGGCGGCGGCGGAGATCAAGAGCTCTTTGATCTCCCCGGAGCCGTCGATCAATCATACTGGAATCAATCACAAGAAAGTCACGTGGTAGGTTGGTGCGGGTGGGGACGACATGCAATTGGTGGGAAGGGAGGGGATGGAAGCGTGAGTCCACGTGGCTGGAGGAGAAGAGTTGGGTTTCAGACCGATGAAACAACATCTCTTCCGCTACTGCAGTTTCCTTTCAACACAACTGCAGTATCAGTCATGGCCATGGTCTACGAAACAAAAGCAATAGAGTTGAGCCATAAAACATGGATGAAGATGGGGAAATGGAAGGGGTTATTAGGAAAGATGAAGACTTGCATGGGGAATGCAAAAGATTGTGTGCTGGTGGTGCTGCTTGATCTGATAGAAATGCAGTCGCAGGATGGGATTATCAGATGGCTTTCTTCTGCTGCAGATGATGAGTTCTCTGTGTTTTGA

>IbDof40

ATGTCGTCGGAGGGCGGCGAGAGACGGCCGGCAAGATTTGAAGGGGTGGCGGCGGAGGCGGAGAAGCTGGCGTGCCCACGTTGCGATTCCACCAACACCAAATTCTGCTACTACAACAACTACAATCTCTGTCAGCCCCGCTACTTCTGCAAGTCCTGCCGCCGTTACTGGACCCGCGGCGGAGCCCTCCGGAACGTACCCGTCGGCGGCGCCACCCGCAAGCCTTCCTCCAAGAAACGCTCTCGTGCTGTCGGGTCGGGTCGATCAGCCCGGTCTCCGGGACCCGTGATGGTTGGAATTAGTGCCGGGTCGGGTCGTGAGGTGAATCTGAACAAGGCGGTGCCGGAGCCGGAGACTGGGAGTTTGAGTTTGGCACCGGTGGGAGGCGGCGGCGTTGAGTTTGCCCCATTGGGTGAGTACGGGCTCGGGCTAGAACAGAGCGGGTTGCATGACGAGTTCGGGCTCGGCCTCGGGCTCTGCGACTGGCCCGCCGAACCAGTCGCCGGAGGCAATGGCGGTGAAGCTGCAAACGTCAATGGAGGCGTGGCTGCCACAAGCTGGAACAACGACGACGCGTGGCAGATTGATGATTGGCCGGAGCTCTCCATTTCTGCACCGGGGAGGAAACCTTGA

>IbDof41

ATGATGGAGGAAAATAGAGGACCTAGTGATTATCATCAATCATTACCGCTAGTGAGGAGGTCGAGAGTAGTGGACTACCATAGCACTCCTCTTCCGCTAATAAAATGCCCTCGTTTGGTCCATGACACCAAGTTTCGCTATTTTAACAACTACAATGTAAATCAACCAAGATATTACTGTCGAACATGCAGACGCCATTGGACCCATGATGGTGCTCAACATGACATCCCTAGAGGTGGTAGGTCTCATAAGGGAAAGAGATCTACTAGGAGGCATGAGAATCAAAGGATTCAATTGGCTCCACCATCACTACCTCAACTCCAACCACTCAATCCATCAACAATTGTGGCTCCACCACCTTTCATTTCACCTATGGCTCCTCCAATGATGACACCATATCACGTTAATGGCGGGTTTTTTCCCCCAATGGTAAAGGAAGAGATAGCTCAACCTCAGCAATGTGAAGCTGAAAGTGGGTACCTCAATTGGGTAAACCCTTGA

>IbDof42

ATGGAGCAAAATAGAAGACCAGGTGATTATCATGCATCATTGCCACTGGTGAGGAGGTCGAGAGTAGTAGACTACCATGGCGCTCCTCTTCCGACAAGAGAATGCCCTCGTTGTCAGTCCAATGACACCAAGTTTAGCTATTTTAACAACTATAAAGACATCCCTAAAGGTGGTAGGTCTCATAAGGGAAAGAGATCTACTAGGAGGCATGAGAATCAAAGGATTCAATTGGCTGCACCATCACCACCTCAACTCCAACCACTCAATCCATCAGCAATTGTGGCTTCACCAGCTTTCATTTCACCTATAGTTCCTCCAATGATGACACCATATCAAGCTAGTGCTAAAAATGGGTACCTCAATTGGGTAAACCCATTGAACATCGTGGACCAAAGCTTTCCACAGCTTACAAATGGGCATTCTGATTCATGTATGGAAAATTATCGTTTGACCAATAGTGACGCTAGCTCGTCTAACATTGTACCATTGGATACATCAATGGGGAAGATTTCCACCACCAATGGAGGCACAAATGTTAGTTGGGATTCATCGTTTGTGGACCTAGATGAATGGCTAGACTTCCCTATTGATTTTAGTCCGTCTGTGTGA

>IbDof43

ATGGAGCAAAATAGAGGACCTGGTGATTATCATCAATCAATGCCGCTAGTGAGGAGGTCGAGAGTAGTGGACTACCATAGCACTCCTCTTCCGCTAATAAAATGCCCTCGTTGTCAGTCCTATGACACCAACTTTCGCTATTTTAACAACTACAATGTAAATCAACCAAGATATTACTGTCGAACATGCAGACGCCATTGGACCCATGGTGGCGCTCAACATGACATCCCTAGAGGTGGTAGGTCCCATAAGGGAAAGAGATCTACTAGGAGGCATGAGAATCAAAGGATTCAATTGGCTCCACCATCACTACCTCAACTCCAACCACTCAATCCATCAACAATTGTGGCTCCACCACCTTTCATTTCACCTATGGTTCCTCCAATGATGACACCATATCACGTTAGTGGCGGGTTTTTTCCCCCAATGGTAGAGCTAGGAAGAGATAGCTCAACCTCAGCAATTGAGGCTAGCTCGTCTAACGCTGTACCGTTGGATGCATCAATGGGGAAGATTTCCACCACCAATGGAGGCACAAATGTTAGTTGGGATTCATCGTTTGTGGACCTAGATAAATAG

**Amino acid sequences**

>IbDof1

MAAANKGDDQMSGDGDKTPAVTRRLKCPRCDSPDTKFCYYNNYSQSQPRYFCKTCRRYWTKGGALRTIPVGGGCRRRKIMKSSPAARYYDIGGLGYLNGVSQPLGGIKIPSYNTSAAALIDQLSGALLAMPQNPPCFNLDQLSPPFSISSPLFDFKFPGTQELGRLAPPIEPVGTVNQDLQWNLQQKILEKQILEIPTPPSEATFESSSSRIAEATEWLFDDDDVTFPLPANQTATNSFGNGNGNASEDDWDELIQAWATNFNEFSALP

>IbDof2

MDSSQWPQGIGVVKGGVVEAPKKPRPEKEKALNCPRCSSTNTKFCYYNNYSLSQPRYFCKTCRRYWTEGGSLRNVPVGGGSRKNRRSSSSISSSSSLASSSSSSKKLPDLAVTPPSFPSHQQNPKGHDLNLAYPPPQINNFGGAGGISEILNLNPNSSAAMEILKNGFSSFMGMPPPVHPDHHHHHSNAAMFLSPGVPLSLDGFCNGYGNLPPQPQPAGTARMFFPNLEDLKPVSTMATDNHQFEEQNRGEGENDGSGYWNGMLGEEEEEEHHGK

>IbDof3

MIQELLAAGNGGLLGGGESNNNAPKLSITDNSSGPLVSSSPTSRSENLRCPRCDSSNTKFCYYNNYNLTQPRHFCKTCRRYWTKGGALRNVPIGGGCRKNKNAIVSASMGKSAAAASTKWKSSFLGGLEPEIPSSKSFLFGPPQNTNHQYPFLSLLRSGQNPNPGANGFILAGGEGQAVGMQELIYQRLMKSSSSSSATNNYNVNCYEHPLALLGNAASSSSSVLSPAILESAPVSAAGEFGYWNPSLSTWSDLPTTGGAYP

>IbDof4

MPEVSKDPAIKLFGKTIQLPDAPAPAPATEMDVDPGGGAASSPPPAEEFSAKDSSCSEAEGGEDEQLQKNENGESEEDEGTVLLEGEEIMDESVDEMRDGLKAPPVDEDDKIIPCPRCDSMETKFCYFNNYNVNQPRHFCKKCQRYWTSGGMTRNLPVGAGRRKSKNSAAHYHHLNAVKSFQSARVDHPERIQHHALKANGKVLTNGSLNGFYKPVELGIPVSYGAGDNGADHSSGSSSSSSTKDDVCKNGLPDMMRQNYHGFAPQLPCFPGPPWPYPSSAVQWSSAVPPPGCFSPGFPMPFYPPAMYLGYTIPCSWNAPWVNRPVSPQQHTSSGPNSPTLGKHSRDDNAVTPASIKEGEPPKDSNPEKCLWVPKTLRFDDPEDAAKSSIWATLGIKHDRVDSIRGGAFKAFKLKDDEKSHASKNCRVLEANPAALSSEQPWVMTLRSYNIFSKIRQLDADFQLFVLRKERFYSIDEQRIGLLAATRGCPSPEVGQRRGRATTKVTPAMYSSLRCTCHKDMFDIKLNIV

>IbDof5

MMEGAPATWKPNVEISPTCPRCGSMNTKFCYYNNYSLTQPRYFCKGCRRYWTKGGSLRNVPVGGGCRKTRRAKSSSIRVVSNHRRGGVFGMSAADNNNPGGSTTSPASAAAGTNGPAPNIDLAAVYANFMNPNPNPQPQPAQIPETTLPNNNDGGGGEGPSFEFSGYPAMLNVDYFVPEANMAPQDGGFVVDEFGNNNNSAAALFQEQFCGESLPPILPPHEELTTSEGWPTHNSDMNMNMNMMFPLHHTISAHHHEPELQGCPNHHTTSSSSLFSIPTTYDSIFRP

>IbDof6

MSSQTLESMMVASAKAQQQEKKPRPADEHAQKCPRCESTNTKFCYYNNYSLSQPRYFCKSCRRYWTKGGTLRNVPVGGGCRKNRRSSSSKARISTAQQDPHTSSPVLTNFPYESPHDLGLTFASLQKQADGNLGLEDHEMAAPMMCNPNNTLSDVLGNYGAASHGFLDSLRATGFLEPPTNGLYHNHSLQYYGTVGEMGMPYDVHMGGGVSTSAAVKQEMYSNGRDNEGDDDNRVLWGFPWQHGGGEGNMNMVVNDVESSRQSSCNIGFGSSWHGLVNSPLM

>IbDof7

MVFSSFPVYLDHSNLHQLQQFAAGYHQQGTGLENPQISTLPAPVGAGQGSIRPGSMVDRARLARMPQPENGLKCPRCDSTSTKFCYYNNYSLSQPRHFCKTCRRYWTRGGAMRNVPVGGGCRRNKRSKKSGGAAAAVSDKQSGGGGAGNSNNINTSTAAHISCPTSCGSAEISGNFSQPPLMAAFQAGLNHFGGFQPPQLVANENGGFGDMSFQLGAEPWRLPASLTAFDTPPPTNLFPFQNEAIEASSSRATPEFPQLTSSVKMEDTQGLNSNSTKQFLSALGNNQFWGGAGGNTWTTGGFSGHLNSSSTSSSTTHLL

>IbDof8

MVFSSFPVYLDHSNLHQLQQSAAGYHQQGTGLENPQISTLPVPAPVGAGQGSIRPGSMVDRARLARMPQPENGLKCPRCDSTSTKFCYYNNYSLSQPRHFCKTCRRYWTRGGAMRNVPVGGGCRRNKRSKKSGGAAAAVSEKQSGGGAGNSNSINTSTAAHISCPTSCGSAEISGNFSQPPLMAAFQAGLNHFGGFQPPQLVANENGGFGDMSFQLGVEPWRLPASLTAFDTPPPTNLFPFQNEAIEASSSRATPEFPQLTSSVKMEDTQGLNSNSTKQFLGALGNNQFWGGAGGNTWTTGGFSGHLNSSSTSSSTTHLL

>IbDof9

MASSSQTLESMLGCTKAEQQEKKAKPSADEQSLKCPRCDSTNTKFCYYNNYSLSQPRYFCKSCRRYWTKGGTLRNVPVGGGCRKNKRSSSRPRTTQDPLFSSLTSTPLMPSLTTTLPYDRPIICNNNPTNNVNPCDVLESYGLLGHLGAGFLQTPATNGLFHNLCYGIGIGIGSEQSNSVEWNGGNGEMMGNSYQDRICSGGVKQEVCYGRDEGESRLNWGFPWLQGGEEGANMVGNSDVISSSRQSWNENGFGPYSSWHGLINTPLM

>IbDof10

MGEHRSVNRNFCTLPITDRNNRSNARVFEQPSRTAGQASAVAVAVASNLNVNSVEASQSRRRQNSILVGEARLTPPPLPVSVSRTSSLHRNSSGEEVAVRQEKKASLSSQPPFWLSGLILLSPGGPVQFLRAIVQYDAVSPAEDNGARLVFIICDFTIPSARFDHFHHCSTRFPADVGGSSQDLNIDVTKPNVSPSRTTDNKSPDFAVEPPPFRSPSSPFLLPKAKGKKGGPGWRGELMEQEALGASGMQPRMWMTGPCRVDARLPDEGNGVGDFHPPLPSARRLRMMEYPGSHPPSRQCPRCHSDDTKFCYFNNYNMNQPRYYCRACKRHSTHGGIQRDIPIGGKSNKGRKSTNRYENKRVQPSLPQLQPPCPQANVAPLAFGPLALPPMVTPYRVENGYLNMVNPLRTIEPPYNSSQNAFQPTSHYDSRSHNLFLNNNDGASSSNPIPLNASTAAPLVDTQILVGVH

>IbDof11

MTISLTPAPAPPQDDGDVSNSVTTPSHDNKNTSGVELMGDKQEVESPVPIAEESVEAVTSSVISEDPPRTQSPDKGTGSSKDIKKDDPSESSDSQEKTLLKKPDKILPCPRCNSLDTKFCYYNNYNVNQPRHFCKNCQRYWTAGGIMRNVPVGSGRRKNKSSATNYHHIMVSENANGAVLAFGSDTPLCKNITTSVLDLVEKPQNCAPNGRYRGSEQAPGCYGGRDTGNEHSNGPSSASIERGGNGAGVTESMWKNFHGFPPQVPCFPGPPPWPCSWNPALNPSSFPVSFYPAPAAPYWSCSPWNVAWISPSSSSDLSAQCSSPMSQTLGKRSREGHILKSSNSSTGEEPLGKTRENRGTTTVLIPKALRIDDSNEAAKSSIWSTLGIKNEKTDSPGASLLKAFSSKADEKNPHVADHSWLMKANPAALSRSLNFHEST

>IbDof12

MAERARLANIPAQDAALNCPRCESTNTKFCYFNNYSLTQPRHFCKSCRRYWTRGGALRNVPVGGACRRNNKRSNTKPPSNSTNNTPTSSTSSPASTTLLGFLPPNFNLPPLLGAQLSDTHFHGGEGVGSLLSGSAAVDPWRLQQASQRQMSLLGGLNPSSYGLYPFQDGSGTMLGQMASNSVMKMEQGNQESRLFSPWSQIFMIPASDEQWNCVADVSANLNSSANM

>IbDof13

MEYPGSPPPPRQCPCCHSGDTKFCYFSNYNKNQPRYYCRACKHHWTHGGIQRDIPIGGKSHKGRKSTNRYENKRVQPSLPQLQPSFPQANVAPLVFGRFGASSDGDTLSR

>IbDof14

MEEIVGTSSGSNNNSSSSSKPSSSSCGERKIRPQKEQAVNCPRCNSTNTKFCYYNNYSLSQPRYFCKTCRRYWTEGGSLRNIPVGGGSRKNKRSSSVSSVSVSVSSANNNGGLAVKKLPDLVPPVPPILHHHHHLHQQQQNPSKIHENHHHHQDLNLGFSQHQDFKTISELIQVPNYESKDGGNDPHRPSSSSSPPSTATHHHHLSALELLTGMTTAATSSSRGLMGSFIPIAPIPDPNNYSSFPLPEFKPSLNFSLDGIGNNNGAYGNHHHHLQGMQETTTASGGGGRLFFPFEDLKSTAAPDGGEQERDNNNPAGESTGGHGFWNGVLGAGGGSW

>IbDof15

MEEIVGTSSGSNNNSSSSSKPSSSSCGERKIRPQKEQAVNCPRCNSTNTKFCYYNNYSLSQPRYFCKTCRRYWTEGGSLRNIPVGGGSRKNKRSSSVSSVSVSVSSAANNNGGLAVKKLPDLVPPVPPILHHHHHHHQQQQQNPSKIHENHHHHQDLNLGFSQHQDFKTISELIQVPNYESKDGGNDPHRPSSSSSPPSTATHHHLSALELLTGMTTAATSSRGLMSSFIPMPPIPDPNNYSSFPLPEFKPSLNFSLDGIGNNNGAYGNHHHLQGMQETATASGGGGRLFFPFEDLKSTAAPDGGEQERDNNNPAGESTGGHGFWNGVLGAGGGSW

>IbDof16

MVFSSVPVYLDPPNWPNQSSALLSAALVAQGQPQPPAAAGGGIRPGSMTERARMAKIPIPEAALKCPRCESTNTKFCYFNNYSLSQPRHFCKTCRRYWTRGGALRNVPVGGGCRRNKRSKGSRSKSPARAAADAPSSSSNNAHRHADILSAAPSHHLPPPPPQIPFLPPLTGFAPPDLGLNFGANEMEFLAGNNSGGGGINLSARFADLFRLQQGITQLPFLSGLDHPAGGMFQFETGIDQYVAAGATDHLGAKPFETGGNLVNVKLEEHNNNNALNLSRNFLGMSGNDQFWSGNNGFAADLSGYASSTGRLL

>IbDof17

MVFSSIPAYLDPANWQQLNHQVGTTTQVPSVPPAAAAAPPPPHGSAGTIRPGSMADRARLANVPMPETALKCPRCESTNTKFCYFNNYSLTQPRHFCKTCRRYWTRGGALRNVPVGGGCRRNKRSGKGSSSGGGGSGNNNNKSPSNSSERQASSSCSGVSTSSIMSGNSGGGLLGGPQIPPLRFMSPLGQLSEHYPTGNDNIGLNINYSGLSAMAGGGGIASLLSGGIEQWRLQTPILGGLDLSQPGLYQFPGGSEAAPSDFLGGETSDTRPKFTSSSMLTTQMASVKMEDHNNNQGSSMARQLLGFQPGNDQWGGGGAAAWSDVSASFSSSSTSNRL

>IbDof18

MDTAQWPQGIGVVENVVETARVAEKKPRPEKEKALNCPRCNSTNTKFCYYNNYSLSQPRYFCKTCRRYWTEGGSLRNVPVGGGSRKNRRSSSSSLSSSLSSSSSTSSKKLPDHSAPSPPCFPLNPNAGQDLNLAYPPPNTSNHGILHHLNPFHIPIPVSDPSTTAVFSSGFMFPLHDVKPAALDGFGNGFVNHVEDSTGARVFFPNLEDLKSGFEQYRAQGETTNGYWNGAHGVAAPPAVAAADGETEQCMHV

>IbDof19

MTPNRAPAAGPQARRRLRGGGGAGVEVPQMRYWTRGGALRSVPIGGGCRKNKKMKSSSSSSSASRLSGDSTGSSGIGGGFKFFNNLSPAMDFQLGGLNFSTLNNVNSPTAAIFEHYSTSSPVIPSNPCFNLDPPPGTAANSVLGFNFSFPSVLKRQGGENTSAGFQEMGSNIHHHPQDNNNNNNNNHLASSIESLSSINQELHWKLQQQRLAMFFGGEHQKEMISTANNSSIPVNEAQMIQKPQPILFQNLDISKPEAFDGKDCGENGSASLATEWLFDNTYAQVNPSATNNTTPGNGNENSTNWNGFQAWSSSNMNHYNSLP

>IbDof20

MPSADTSDQRRAAKQQQVGAPPPEPEHLRCPRCDSTNTKFCYYNNYNFSQPRHFCKSCRRYWTHGGTLRDIPVGGGSRKNAKRSRTIPSSSSGAAAATNAAAFPAHDFRHAAAPSSFLFPLGADHAGSVPFPADMVKPGLNVCGSFTSLLNTQGPGLLALGGFGLGPTIDDMGYGFSRAVWPFPGVPEAAANGGTAAVLSGGPWQLSTGENAFVNGDCFGFARSCNFYPGNRRPVIAGNINGRACSKVNRRMSGREVHLRLCSLIDDSLRPYVEVMPTSFNKENVKELLIALSQVCSQIKRWTVEFASDSDSDGVESDSDAEVTGNSAGVGLVPGSETESHYSLAKAISVLMGLLAIEYPYVQHLVGNILVAISDFIVASGSSSDEFMQLLSLLKLAAASSFPSSTGCIMAEAKNSCCNSAASVSPLSPHHKSAKWLTVAVIIQVLRRILKNLKQDADDHFFKLYLGVTNSFISNMPWDLMDQVFVSQTSKALGDPIADGLLQFQPAKSKSAIMFLGNFIQLLCSLVEKDIPIEAVADDLDKHPLICEIRNILPRLLGWCLGNLQNSDVYISKYYKHKLLILMIRLSFKIQLDCSVLLSWLHLIHLYFQDLLCLPIAGLESDKDKYLEGSPFWEDIFDAAKENISSRHLHRLAIFLFLRCSFGLVNMEKSGQNCASTDLNSHSDPNLDIECCTQSKGLQELHEWLQRLFNDVFLDHEKSAESIVSFQFSFIQLYMHEDDILFQMLLQLLCVPLCSEKWSVKEGNPSAYDNAYSIVLHLFNPVHLFHIFLAEIHYDHQVLLDYLISKDTGASSAEYLLRCLSMVFKSWSLFTNFAWSGKKEIQLYQKRRKVSLDHLSFKVEVSTPVTDGRCSPVERDQKKGKASGVIDCRTERLPFQHATNCLLELKTSIENLHHKNLFPYNPKVLLQRCLVNVSGALPKAVGSESKYLVEEMAKMNVSA

>IbDof21

MELSNPTQTRALTRPVRSPCERASLCFATLEVGKKEKKWARMSDSSKDPAIKLFGKTIQLPEIPASTAPATEGSCPDDAPGDDSSARHGSGSGNDPAEDGGGGEEDEHLQKEPESFYLGFIVSLAFSTFDPIKKGGKLDESKDDESLMAEELTDQTSEMETDELKTPSTNKDCGAMKKSEVEEQGETGNSQEKALKKPDKILPCPRCNSMETKFCYFNNYNVNQPRHFCKNCQRYWTAGGTMRNVPVGAGRRKNKNSRFPLPPCDHLGALSECPSRPSEWNPASYDQSQWHPSYNGSINGFHKLEELGVPVPHGAGDNGDDCSSGSSVTAASTKDESVKNEVPGRQNCPNFAPQLPCFPGAPWPYPLTAVQWSSAIPPPGYCPPGFPMPPPLAPQHHMPSTSGPNSPTLGKHSRDENMLKPVNSEGEDPKKESNPDKCLWVPKTLRIDDPEDAAKSSIWATLGIKNDKADSVGSGLFKAFQSKDDEKTDASENSTVLQANPAALSRSLNFHENS

>IbDof22

MGEGKDPGITLFGKKIPLPLRVVVFAGDESAGGDSGGVNSPDESNSGSERVRCFDDENIEESKREDEMDQLPGEASETISEEKDDDEDEDQNMDGKESCKALQESGSNTETPSTDGNSPTAKSTKTEDDQTETDSSKEKTLKKPDKILPCPRCNSMDTKFCYYNNYNVNQPRHFCRSCQRYWTAGGSMRNLPVGAGRRKNKSSASHCHITISNANVSDALHAMQAEPPNGFHYPAHYKPNGTVLSFSPNSSLRECMGSVLNPGETKSPNGTQNGFSKPDTRNSSYKTGNDCSSKSSVINSNSKAEGSKNAPQDTTVMQNVHAFPSPVPYLPKVPWNAAAPLPPIFPSGAPVTFCPATYWNYCLPGPWSLPWLTPPSPTVSQKSSISSPNSSLGKHPRDGDLLEPNNPKGKESSEQKSPERRILVPKTLRIDDPDEAAKSSIWATLGIKYDSISKGSMFNALEQKKR

>IbDof23

MGLTSLQVCMDSSVDWLQGTIHDEGGMDSSSSPSGVDILTCSRPLIERRLRPPHDQALKCPRCESTHTKFCYYNNYSLSQPRYFCKTCRRYWTKGGTLRNIPVGGGCRKNKKASSSSNNSSSSSSSANKKSSNDHNQFPHPPPPLYHHHQNLGQSSSSTSSPGSAGINPTDLHLSFPAEMQFSHHFTNFHLAPHTGFLGIESPTPIDFMEGKYDSGFVMGGATCTTPPPPSRNQDFLGNNNGDFGMMAQTTFPTSISPQFGMPIDGNSVGPTLMLPYDHHHHHGDHHQFNNAMHHHNHQDVKPNPKLLSLEWHDQGCSDAGKDSFGYLNGVGSWTGLMNGYGSSATNPLV

>IbDof24

MGLSTKQVSGGDHHQHGIIEWENPQNPGGSTRLDLPTVQAQSVQPPPQKAEPLKCPRCASENTKFCYYNNYNKSQPRHFCKGCKRHWTEGGTLRNVPVGGSRKNKRLKTPPNANAVTPAVKSSSPAAATCDNQNPVFSGENKNHAPSLFFPAAIQADPVIGGAAAAFNIGSAPMVLHDDQDTTKLLPFSFSSFFDPISCSIPSFNTQSSSNNAYSDIIVGELDNVEESTITTVMPFTSSAVISQPWDMANYWSLSEIDSLVSADLNIPWDDLEIKPKGK

>IbDof25

MVGSNCDEKMAVISSTANEWPIHQKNQIDGRSLMGSRVMEKPMNGQDQAMPPPPPPNQQPLKCPRCDSSNTKFCYYNNYSLSQPRHFCKACKRYWTRGGTLRNVPVGGGCRKNKRIKRPSAPSVSSSSATQDHHISAPSSSAPNLQVQAQMDHHINPLLYGLPTSTPSELGLSFPRLFTSRVSNFEGTNIPGYDHHHHMGLGFSSSSNSGLLGGENGFPNNNTSNNSLLSSFHGGLSCSSSSSSSSSTLASLIASSLHQGANNNGFHGLSPYGEMQVGNGNMGPKEVKMEGNWNNNNNNNTNSLSSHHYQIMNQTAQINTSSSDNPSLPWNNGGSWFDPSNMGSSVPSIL

>IbDof26

MAEVEDSHSAQGIKLFGATIITVQEKKSEDEKSEVKGDDDHGSTADNQKRPDKVIPCPRCKSMETKFCYFNNYNVNQPRHFCKGCQRYWTAGGALRNVPVGAGRRKNKPPCRGMIDGLSEGCLFDASGLLQNLDFDGAVVEEWYAAEQGGAGVGGFRNLLPAKRRRKISAGQPC

>IbDof27

MAEVEDSHNAQGIKLFGATIITVQEKKSEDEKSEVKGDDDHGSTADNQKRPDKVIPCPRCKSMETKFCYFNNYNVNQPRHFCKGCQRYWTAGGALRNVPVGAGRRKNKPPCRGMIDGLSEGCLFDASGLLQNLDFDGAVVEEWYAAEQGGAGVGGFRNLLPAKRRRKISASQPC

>IbDof28

MRPMEENQEIVPIIGNTRGERKGSIRFQKDQVLNCPRCNSANTKFCYYNNYSLSQPRYFCKTCRRYWTAGGSLRNIPVGGGSRKNKKPLKKVADLVPPVPISGDHQSSSKIHGGEGHHQDLNLGGQLSAMELLTGFSTSIGLSTAPFISDPNLLYSQAGISLPESKQCLKICLDGIGNLQPMQDTTTNTRFLLPSDQDLRSTASDDNGVLRGDGGGESW

>IbDof29

MLSSESSPMLDCHSIRPILMDRKWKPNIELAPNCPRCASTNTKFCYYNNYSLSQPRYFCKGCRRYWTKGGSLRNVPVGGGCRKTRRSKSARQAAEQHRSNAHHAPAGALDNGSAGAEEIDMAAVFAKYLNQGDLSPSGATSSTSASMALSSLDSESQVDELLLLDYQEDPSPLFLDQAVELQESSPQASINVQEHLLDYNQSALDLQALLEDDQWPNFAWQQPMIQQQDLGTFFGDNDLVYPTKASTTLPNNDVWGSFDLSGCEILPRP

>IbDof30

MLSSESSPMLDCHSIRPILMDRKWKPNIELAPNCPRCASTNTKFCYYNNYSLSQPRYFCKGCRRYWTKGGSLRNVPVGGGCRKTRRSKSARQAAEQHRSNAHHAPAGALDNGSAGAEEIDMAAVFAKYLNQGDLSPSGATSSTSASMALSSLDSESQVDELLLLDYQEDPSPLFLDQAVELQESSPQASINVQEHLLDYNQSALDLQALLEDDQWPNFAWQQPMIQQQDLGTFFGDNDLVYPTKGSTTLPNNDVWGSFDLSGCEILPRP

>IbDof31

MSTEVKDPAIKLFGKTICLLRDDTLPCSIAADQPAKRSRSSITTTSRDSKNTSGEVSESQHQDDECRNPSGEDSVGAETSSRTSDDDKAAQNPDKETQCAAGKDVKKDDESETSDSQDQKNLKKPDKILPCPRCNSKDTKFCYYNNYNVNQPRHFCKNCQRYWTAGGSMRNVPVGSGRRKNKASATNFRHIMVSDALHAAQFASLKPNGTVLTFGSDRAVSNESIIPNAWNSAPPPLYGVLAPWSGRWISAPASPDPSTPTSPRRLKMEDQAIWSTPGGGGGSLFKAFNSKDGERDRDRNHSLSKGFTRVSFKAVHVRRVWRQEKKVLVMKKFNIILTVPEFSPDSAGVQTPPVSLLNLTFRFPAAPLPLFRFHFLLQIPHNFVESNRTYALGACEVELCSGHQFFVQQSWCPQHISIPLKVQQAYGLMGRNRLPLKRLTSANRRIAYSRRKKGLMKKARELSVLCDVEVFLVTFSPGGKPTVFITENSSIEDVIEKFAQLKPEERAKEKLECLEVVKKACKKFDHHVDIGELFYPGDLSDEDLTSLADSLRTRFSDTQNRLSHWMNIDKISNTDQLGKMEESLINSLQDIQRHKNGLLAQQLKLQCSDELQHVTDFPIGMGTSQVLQPSSWTQNGINQNINFDKDLNLTQGYHRCIVSRLPECSFESYTNLFCFDKEVEVTRPSGEDRTNPLLDYEHMQHYFQFQSAEENLVSCFPYLSPGTTEFPSFDVTDKSLEELFENSGNHFSDRFLSSYGVDSQLLRPLDASFHEYTAKYIKILQGLMGQQKCWAILLNCPDFNLV

>IbDof32

MMEGAPATWKPNVEISPTCPRCGSMNTKFCYYNNYSLTQPRYFCKGCRRYWTKGGSLRNVPVGGGCRKTRRAKSSSIRVVSNHRRGGVFGISAADNNNPGGSTTSPTSAAAGGTNGPAPNIDLAAVYANFMNPNPQPQPAHIPETTLPNNNDGGGGGGADPSFEFSGYPAMLNVDYFVPEAAMAPQDGGFVVDEFGNNFNNSGAAMFQEQFCGESLPPILPPHEELTTSEGWPTHNSDMNMNMMFPLQHTISAHHHEPELQGCPNHHTTSSSSLFSIPTTYDSIFRP

>IbDof33

MMEGAPATWKPNVEISPTCPRCGSMNTKFCYYNNYSLTQPRYFCKGCRRYWTKGGSLRNVPVGGGCRKTRRAKSSSIRVVSNHRRGGVFGISAAADNNNPGGSTTSPTSASAAAGGGTNGPAPNIDLAAVYANFMNPNPNPNPQPQPAHILETTLPNNNDGGGGGGADPSFEFSGYPAMLNVDYFVPETAMAPQDGGFVVDEFGNNINNSRAAMFQEQFCGESLPPILPPHEELTTSEGWPTHNSDMNMNMNMMFPLQHTISAHHHEPELQGCPNHHTTSSASLFSIPTTYDSIFRP

>IbDof34

MLGCTKAQQEKKPRPADQQALKCPRCESTNTKFCYYNNYSLSQPRYFCKSCRRYWTKGGTLRNVPVGGGCRKNKRSSSSASSRSRSQDQSLCSSPVPLPSLAGLPYEASDLSLALARLQKQANLGVGEHEMGMMCNPNNTPYDIVLGNHHHGFLETAANASFHNMYYGNINVGGEMGIPYEEHHLGGGGAAAAVKQEMCSAREEGESSRVLWSFPWQQVGADHQGNNNMGDQIDSSKQNWYGYGNGFGSSSSWHGLLNSPLM

>IbDof35

MQDVHPISGGGARMFAGGGDRRLRPHHHQTAELKCPRCDSLNTKFCYYNNYNLSQPRHFCKSCRRYWTKGGVLRNVPAGGGSRKTKRSKQKSVDCRSGGHLSLIPIPVARVLASRAAAAAAAAAELGNFTTLMTSSDGPSSLVEFTNAAAAAADTFRLPHSPKAQWGPQPKMDGEDGKMQDITAAGFLDDATQSRRSNGGLSPLDWLIGGGQGHGLFDLTGAVDQSYWNDDHTLNYLPL

>IbDof36

MSKLPPADEDSPSPKTSKTENDQTETNNSQQKTLKKPDKILPCPRCNSMDTKFCYYNNYNVNQPRHFCKSCQRYWTAGGTMRNVPVGAGRRKNKNSASHCRHITITEALQAARIDVPNGFHHPTYKPNGTVLSFGPESPLCESMASLLNLADKKVPNGMPNGFYKHEQGNSPNKVGENGDDCSSVSSVTTTSSMAGGKNPPQEAVMPNINGFPTPVPCLPGVPWPFPWNAAVPLPAICPPGFPMPFCPAPYWNCAVPGPWSLPWLAPPSPTANQKTSSSSPNSPLGKHSREGELLTPNNPEAKESSEQKLWKFSFGVGFSRPCNQKVMKRVTQQLLLQHCRLILQHYLDLSASRKAPKVGLGRLKAPTVGYKTCRDETLNSLKATASS

>IbDof37

MVFSSIPAYLDPSNWQQQQLNHHLQGGSSGIPTPHLAAAAPPPPVVGGGGGSIRPGSMAERARLANMPTPEVALRCPRCESTNTKFCYFNNYSLSQPRHFCKTCRRYWTRGGALRSVPVGGGCRRNNKRSGKGGGGGGASSSSSKSTSSTTTASNDRQQSNNSGTVSAVSGPTTAHNLLGLSPQIPQLPFMSSSPIPQLSDHHYAAGISPAADNLIGGGGGGDNLLGGGGVSPTSFIGGAPFRPRISSAMLAQLAAVKMEDTNNNNHTINNQESSLPRQILGINNNIINPGGNEHWSGDNNGGGGGWSDISASFSSSSTSNTHL

>IbDof38

MMNSYQTDGKSLMASSSSSSPSSSTAARVMEKPISQSEQQPLKCPRCDSSNTKFCYYNNYSLSQPRHFCKACKRYWTRGGTLRNVPVGGGCRKNNKRIRRSSSSSSSPAAIDVVSSSSTSPAVSSAPLQIPAPQTKPQINPLFYGLPINPNSELNPPFPRLFGSRVSSSESFDNSNHDSSMISSSPGLLGGHGENNGFKFVSSLIIKSSSFKLPTSSLEQRKFNASGDITNRNFPGMPSYNGESVMAGNMILENEEFLKGESQNRLNWNGGVPMNPMNNHNLHTEGVVNFNYSTPSSDPSFPWNGGWMEPSLI

>IbDof39

MQDIHSIGGGGGGRLFGGGGDRRLRPNQHHNLPALKCPRCDSLNTKFCYYNNYNLSQPRHFCKSCRRYWTKGGVLRNVPVGGGCRKSKRSKPKSTASASASASTPADASQECKSNSHSSSESSSLTGAGTASAAAEVASTRYWNLLEHDHSLQRTLPIRVRQYGRHFPDVPDSTEPARAGRSLAATGKIADQNFNPPEITAAGFLDQTSQIDFPSLNQQQSRESNAGLPPLDWQPGGGGDQELFDLPGAVDQSYWNQSQESHVVGWCGWGRHAIGGKGGDGSVSPRGWRRRVGFQTDETTSLPLLQFPFNTTAVSVMAMVYETKAIELSHKTWMKMGKWKGLLGKMKTCMGNAKDCVLVVLLDLIEMQSQDGIIRWLSSAADDEFSVF

>IbDof40

MSSEGGERRPARFEGVAAEAEKLACPRCDSTNTKFCYYNNYNLCQPRYFCKSCRRYWTRGGALRNVPVGGATRKPSSKKRSRAVGSGRSARSPGPVMVGISAGSGREVNLNKAVPEPETGSLSLAPVGGGGVEFAPLGEYGLGLEQSGLHDEFGLGLGLCDWPAEPVAGGNGGEAANVNGGVAATSWNNDDAWQIDDWPELSISAPGRKP

>IbDof41

MMEENRGPSDYHQSLPLVRRSRVVDYHSTPLPLIKCPRLVHDTKFRYFNNYNVNQPRYYCRTCRRHWTHDGAQHDIPRGGRSHKGKRSTRRHENQRIQLAPPSLPQLQPLNPSTIVAPPPFISPMAPPMMTPYHVNGGFFPPMVKEEIAQPQQCEAESGYLNWVNP

>IbDof42

MEQNRRPGDYHASLPLVRRSRVVDYHGAPLPTRECPRCQSNDTKFSYFNNYKDIPKGGRSHKGKRSTRRHENQRIQLAAPSPPQLQPLNPSAIVASPAFISPIVPPMMTPYQASAKNGYLNWVNPLNIVDQSFPQLTNGHSDSCMENYRLTNSDASSSNIVPLDTSMGKISTTNGGTNVSWDSSFVDLDEWLDFPIDFSPSV

>IbDof43

MEQNRGPGDYHQSMPLVRRSRVVDYHSTPLPLIKCPRCQSYDTNFRYFNNYNVNQPRYYCRTCRRHWTHGGAQHDIPRGGRSHKGKRSTRRHENQRIQLAPPSLPQLQPLNPSTIVAPPPFISPMVPPMMTPYHVSGGFFPPMVELGRDSSTSAIEASSSNAVPLDASMGKISTTNGGTNVSWDSSFVDLDK
